# Osseointegrability of 3D-printed porous titanium alloy implant on tibial shaft bone defect in rabbit model

**DOI:** 10.1371/journal.pone.0282457

**Published:** 2023-09-08

**Authors:** Hung Do Phuoc, Phu Nguyen Hoang, Sam Yang, Darren Fraser, Vu Thua Nguyen

**Affiliations:** 1 Department of Orthopaedics and Rehabilitation, University of Medicine and Pharmacy at Ho Chi Minh City, Ho Chi Minh City, Vietnam; 2 Department of Orthopaedics and Traumatology, Cho Ray Hospital, Ho Chi Minh City, Vietnam; 3 Manufacturing, Commonwealth Scientific and Industrial Research Organisation, Clayton, Victoria, Australia; University of South Carolina, UNITED STATES

## Abstract

Previous studies have demonstrated the ability of osseointegration of porous titanium implants in cancellous bone. Our study was designed to (i) investigate the ability of bone ingrowth into 3D-printed porous titanium alloy implant on the cortical bone of rabbits using CT-scan and histology, and (ii) to identify the consistency of the radiology information between clinical Cone Beam Computed Tomography (CBCT) and Micro Computed Tomography (μCT) in the evaluation of bone ingrowth. The porous titanium alloy implants were 3D-printed employing the Electron Beam Melting (EBM) technology with an intended pore size of 600 μm and porosity of approximately 50 percent. Each implant was inserted into tibial diaphysis in one rabbit and its pores were classified as contacting bone or non-contacting bone. Depending on the time of explantation, the rabbits were divided into two groups: group 1 consisting of 6 rabbits between 13 and 20 weeks and group 2 consisting of 6 rabbits between 26 and 32 weeks. Tissue ingrowth into the non-bone contacting pores were evaluated by CBCT and histology. μCT was used to further investigate the bone ingrowth into four implants (two from each group were randomly chosen). The CBCT detected the present of tissue with bone-like density in both bone-contacting pores and non-bone-contacting pores of all implants. The μCT analysis also supported this result. All the bone-like tissues were then histologically confirmed to be mature bone. The analysis of CBCT data to assess bone ingrowth in porous implants had the sensitivity, specificity, positive and negative predictive values of 85, 84, 93 and 70 percent, respectively, when considering μCT assessment as the gold standard. Fully porous titanium alloy implant has great potential to reconstruct diaphyseal bone defect due to its good ability of osseointegration. CBCT is a promising method for evaluation of bone ingrowth into porous implants.

## Introduction

The management of bone defects caused by trauma, infection and tumor resection remains the considerable clinical challenge in orthopedic surgery. The condition may result in physical impairment, psychological influence, and badly economic impact on the patient’s life. A critical-sized bone defect is defined as one that would not heal spontaneously in spite of surgical stabilization and requires further intervention. In adult patients, general guidelines for critical bone defect in the literature include defect length greater than 2 cm and more than 50 percent circumferential loss of the bone [1]. The treatment requires not only the surgeon’s experience but also the patient’s strict adherence to a specific postoperative program. The current management includes bone grafting, induced membrane technique and distraction osteogenesis. It might involve multiple surgical procedures, prolong hospital stays and have high risk of complications such as donor site morbidity, infection, delayed/non-union and refracture [1–3]. In addition, several types of bone defects have not been effectively reconstructed because the requirements of the unique anatomical shape were not met.

Titanium and its alloys have long been used in orthopedic and dental fields because of their outstanding characteristics such as excellent biocompatibility, osseointegration, high corrosion resistance, high fatigue strength, good strength to weight ratio and fracture toughness [4–6]. Although Young’s moduli of titanium alloys are lower than those of stainless steel or other metallic biomaterials, they are still much higher than those of human bone [7, 8]. The mismatch of elastic modulus between the titanium implant and the bone would lead to stress shielding effect, which then causes bone resorption and peri-prosthetic loosening as a result [9–11].

With the advance of metal additive manufacturing, various titanium porous structures have recently been developed to achieve the mechanical properties similar to those of bone, particularly the yield strength and the Young’s modulus [12]. Moreover, bone ingrowth into the pores, as observed in rabbit models, enhances the fixation between the implant and surrounding bone [13–16]. However, a majority of studies have focused on the investigation of bone ingrowth at the epiphyseal and metaphyseal zones, which are mostly composed of cancellous bone [17, 18]. The process of bone ingrowth into implant might take place in a different way at the diaphyseal zone where cortical bone mostly presents. While cancellous bone in metaphysis normally heals by intramembranous ossification, cortical bone in diaphysis heals by a combination of endochondral bone formation and intramembranous ossification [19, 20]. Jan Wielding et al. [21] performed a study on bone ingrowth into two types of open-porous scaffolds at metatarsal diaphyseal segmental bone defects of sheep. The scaffolds were coated and filled with calcium phosphate to enhance bone regeneration and stabilized by plate and screw. That can lead to misinterpreting the osseointegrability of the original 3D-printed implant. The study [21] also did not evaluate the bone ingrowth into the porous implant fabricated by selective laser melting technology due to the low resolution of CT-scan data (230 μm). In addition, they [21] did not perform histological evaluation of the new bone formed within the pores of the implants. To gain better insight into the osseointegration between porous titanium implants and cortical bone, in-vivo study employing rabbit model was carried out. In this work, we performed histological and radiological (CBCT and μCT) assessments to evaluate the osseointegrability of 3D-printed porous titanium implanted on tibial shaft bone defect in New Zealand white rabbits.

In the following sections, the materials and methods used in this study are described in details. Results and analysis are then provided. Finally, some conclusions are drawn from this study.

## Materials and methods

### Ethical statement

This study was carried out in strict accordance with the recommendations in the Vietnamese Law on animal husbandry, the Directive 2010/63/EU of the European Parliament and of the Council of 22 September 2010 on the protection of animals used for scientific purposes and in the AVMA Guidelines for the Euthanasia of Animals. The study was conducted following strictly the ARRIVE guidelines. The protocol was approved by the Ethics Committee for Biomedical Research of Cho Ray hospital, Vietnam (registry number: 673/BV-HDDD). The hospital’s Ethics Committee for Biomedical Research has jurisdiction to oversee animal research. All surgery was performed under the combination of Ketamine and Xylazine anaesthesia. All euthanasia was carried out with the induction of Ketamine and Xylazine anaesthesia followed by overdosing intravenous potassium chloride. All efforts were made to minimise suffering.

### Study design

The animal study employing 12 mature New Zealand white rabbits was conducted to assess the bone ingrowth into the 3D-printed porous titanium alloy implant inserted in the rabbit tibial shaft defect. In each rabbit, a 12 mm x 3.6 mm rectangular bony window of left tibial diaphysis was created and filled with the implants of the same size. The rabbits were then randomly divided into two groups (n = 6). After a period of time (group 1: 13–20 weeks; group 2: 26–32 weeks), the animals were euthanised before the procedure for explantation of the tibia and the implant was carried out for CBCT and histologic investigation (a total of 12 CBCT and 12 histologic investigations). Four of the specimens—two obtained from each group—were further investigated using μCT. In this study, the wide range of explantation time was chosen to allow for observation of the progression of osseointegration in porous implants.

### Experimental animals, housing and husbandry

Twelve mature male New Zealand white rabbits weighing between 2.50 kg and 3.20 kg were obtained from the Pasteur Institute of Nha Trang, Vietnam and received a period of acclimation between 14 to 16 days before surgery. The rabbits were housed separately in the standard cages with free daily access to water and 100 grams of food until they were euthanised.

### Implants

**Fig 1:**
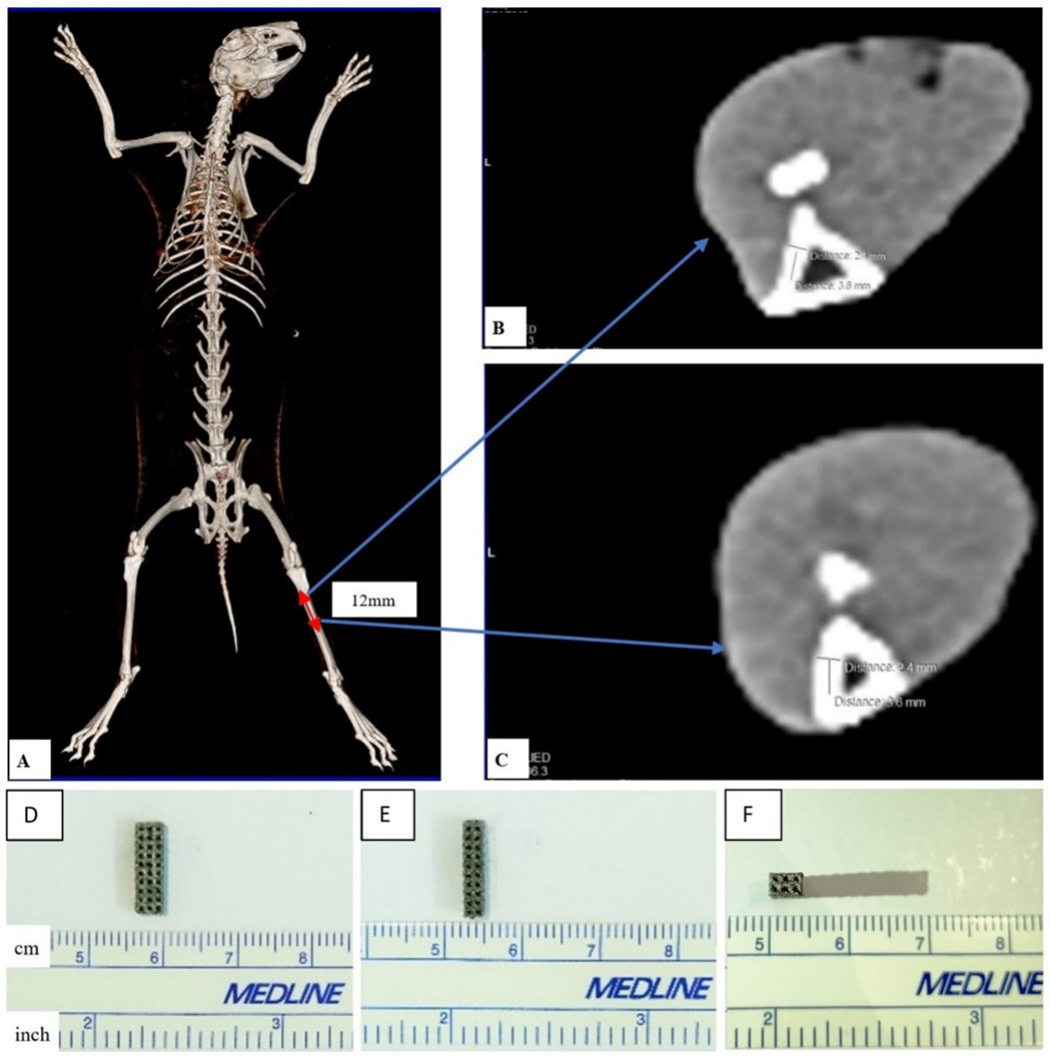
The CT-scan of the smallest rabbit and illustration of 3D-implant before implantation. (**A**) The smallest rabbit weighing 2.50kg before implantation (rabbit number 9). Its upper (**B**) and lower (**C**) axial slices illustrates the dimension of lateral cortex of the tibia. The illustration of 3D-printed porous Ti6Al4V implant before implanatation in 3 planes: (**D**) Coronal view, (**E**) sagittal view and (**F**) axial view.

A computer-aided design (CAD) software was employed to create the lattice structure that consists of cubic units suitable for the powder bed fusion processes without complex support structures. The implants were designed to have a porosity of 50 percent with pore size of 600 μm since this size is reportedly favorable for bone ingrowth [14, 22]. Based on the measurement of the lateral wall of tibial shaft bone of the smallest rabbit (Fig 1A–1C), we designed the implants having dimensions of 3.6 mm x 2.4 mm x 12 mm so that they can be used for all other rabbits. We employed finite element analysis (ANSYS) to estimate the equivalent elastic modulus of the design. The implants were additively manufactured using Electron Beam Melting (EBM) system Arcam Q10 (Arcam AB, Sweden) and Ti6Al4V ELI powder having particle sizes ranging from 60 μm to80 μm. The implants (Fig 1D–1F) were then ultrasonically cleaned to remove the residual powder, conventionally sterilized in a low temperature hydrogen peroxide gas plasma (STERRAD) system and ready for implantation.

### Anesthesia and surgery

Surgeries were performed under sterile conditions. The rabbits were weighted right before the operation to allow for accurate estimation of drug doses. Preoperatively, a rabbit received 30mg/kg of Ketamine in combination with 3mg/kg of Xylazine intramuscular injection to initiate anesthesia [23, 24]. The skin of the left hind leg was shaved enough, and then disinfected with 10 percent povidone iodine (Betadine solution) from the thigh to the ankle. The midline incision on the middle third of the leg was made and the tibia was exposed after retraction of lateral muscle compartment. We used a dental burr to create a bone defect on the lateral wall of the tibia with the size approximately 3.6 mm x 12 mm. Surgical field was irrigated with saline solution to wash out any bony debris and blood clot. The implant was inserted into the defect using press-fit fixation (S1 Fig). Afterward, the wound was closed, using layered closure technique and draped with sterilized gauzes.

### Postoperative follow-up and euthanasia

**Fig 2:**
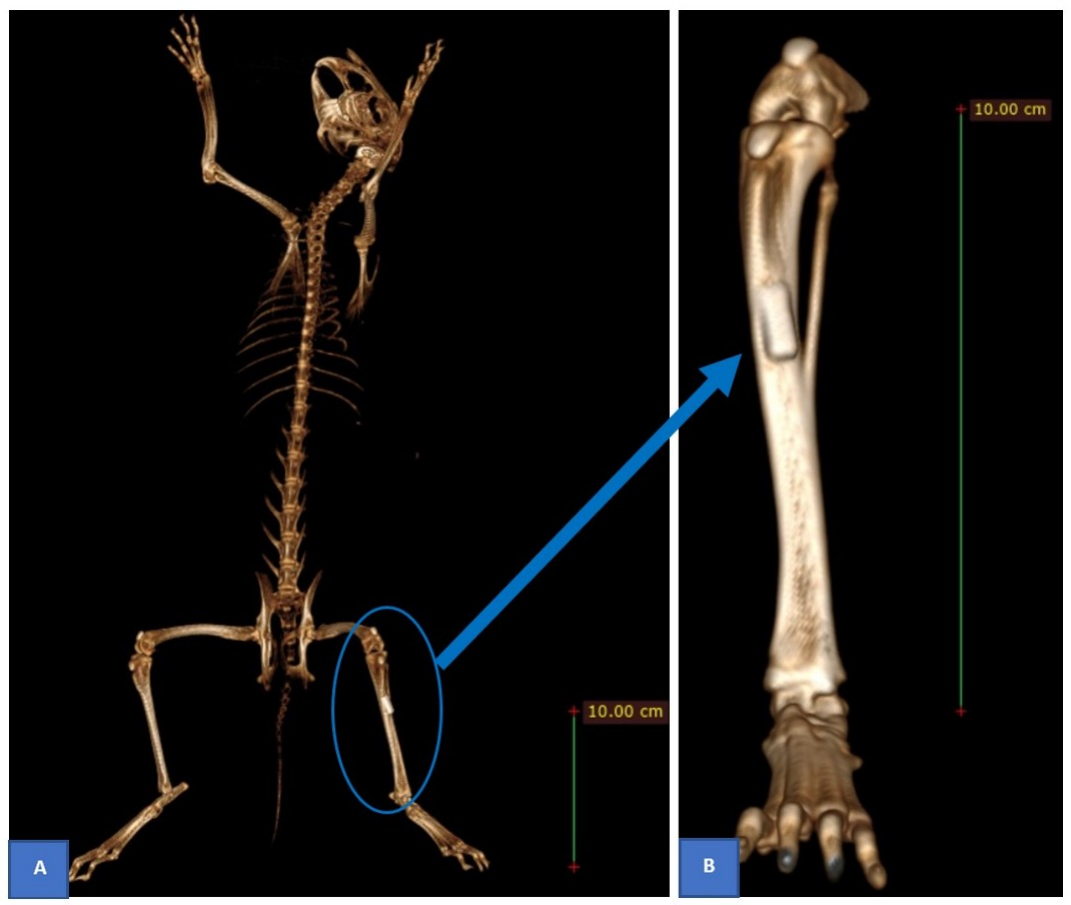
Postoperative control CT-scan reconstruction was obtained to confirm the position of the implant (sample 2). Full body (**A**) and implant position on the left hind leg (**B**).

The first two rabbits—one from each group—that had undergone surgery subsequently received postoperative conventional CT-scan with the low resolution of 1 mm of slice thickness to confirm the implant position (Fig 2). The activities of the surgical hind leg were followed up to detect any signs of fracture or infection. We also noted the first date of hind legs weight bearing as the first date when the rabbits could stand on both their hind legs during the postoperative period. The rabbits were then euthanised within a period of 13–20 weeks (group 1) or 26–32 weeks (group 2) by overdosing intravenous potassium chloride 10% [25] after inducing general anesthesia.

### Postmortem sample acquisition and analyses

**Fig 3:**
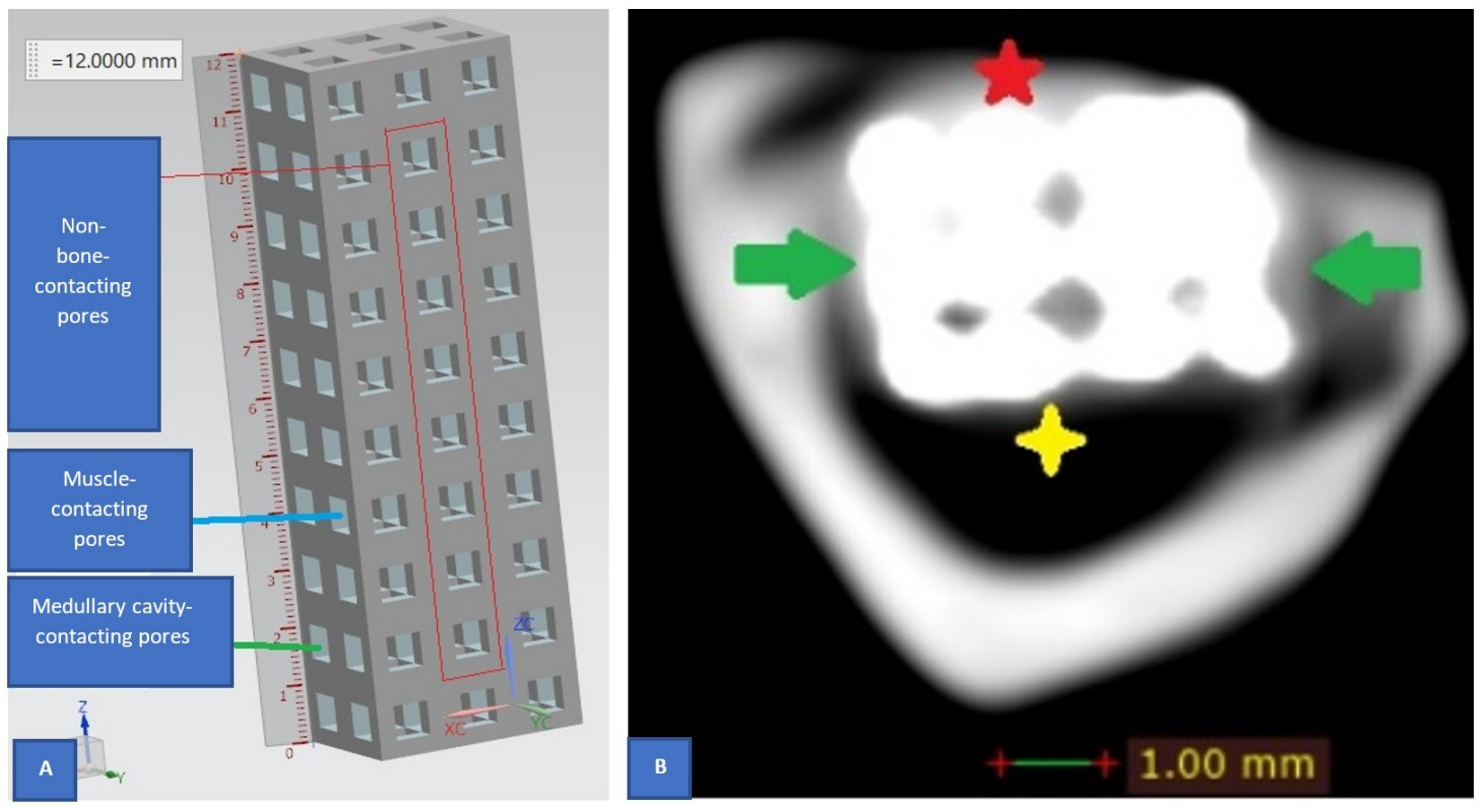
(color) Illustration of pores classification. (**A**) The non-bone-contacting pores are pores situated in the center of the implant (inside the area marked by red rectangle) whereas the bone-contacting pores situated at the periphery (outside the area marked by red rectangle). (**B**) The axial CBCT slice illustrates the bone-implant specimen and another classification of pores: the muscle-contacting pores located next to the lateral muscle compartment (the side marked with red star) of the hind leg and the medullary cavity-contacting pores (the side marked with yellow four pointed star). Green arrow indicates the bone-contacting pores.

**Fig 4:**
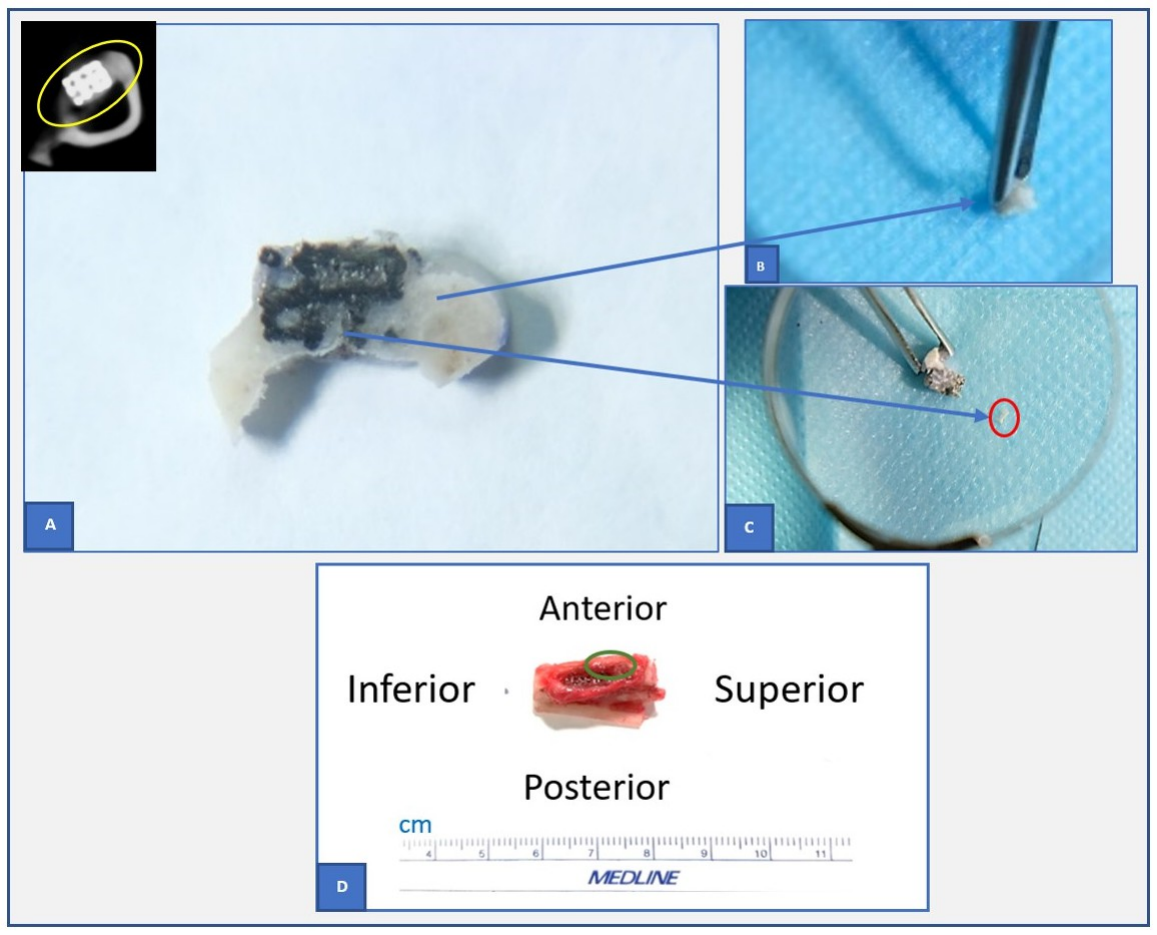
(color) The illustration of extracted specimens used for histological evaluation. Extraction of bone-like tissue (**A-C**) and membranous tissue (**D**) used for histological evaluation (the tissues in the areas marked by red and green circles were collected). (**A**) The sample of the bone-implant specimen and the corresponding CBCT slice shown at the top left corner of (**A**). The yellow circle indicates the actual portion in (**A)** getting from CBCT data.

A tibial segment containing the implant was cut by a dental diamond disc tool (Adenta, Switzerland). The segment was then observed and the membrane covering the implant surface was extracted for separately histologic evaluation. Two specimens obtained from each rabbit were then individually fixed in 10 percent phosphate-buffered formalin (pH 7.25) before the specimen of the tibial segment being scanned employing clinical CBCT. The CBCT data were then processed with Radiant DICOM Viewer 64 bit (Medixant, Poland) to assess tissue ingrowth into pores and identify the location of pores with bone-like tissue among the non-bone-contacting pore (Fig 3). Using a dental diamond disc (Adenta, Switzerland) the implant was divided into two halves at the level where bone-like density was most likely presented in the non-bone-contacting pores. The piece of bone-like tissue and the membrane were then stained with hematoxylin and eosin for further histological evaluation (Fig 4). In addition, four specimens (two obtained from each group) were randomly chosen for further investigation employing μCT before the bone-like tissue is collected.

### CT-scan

The first two rabbits—one from each group—that had undergone surgery subsequently received postoperative conventional CT-scan (SOMATOM, Siemens) with the low resolution of 1 mm of slice thickness to confirm the implant position. After explantation of the 12 tibial—implant specimens, preliminary X-ray CT scan for samples were conducted using a clinical CBCT scanner (Promax3D Plus) that was set with an effective pixel size 100 μm, at X-ray tube voltage 90 kV and current 12 mA. The artefact removal parameter was set to high in CT slices reconstruction.

**Fig 5:**
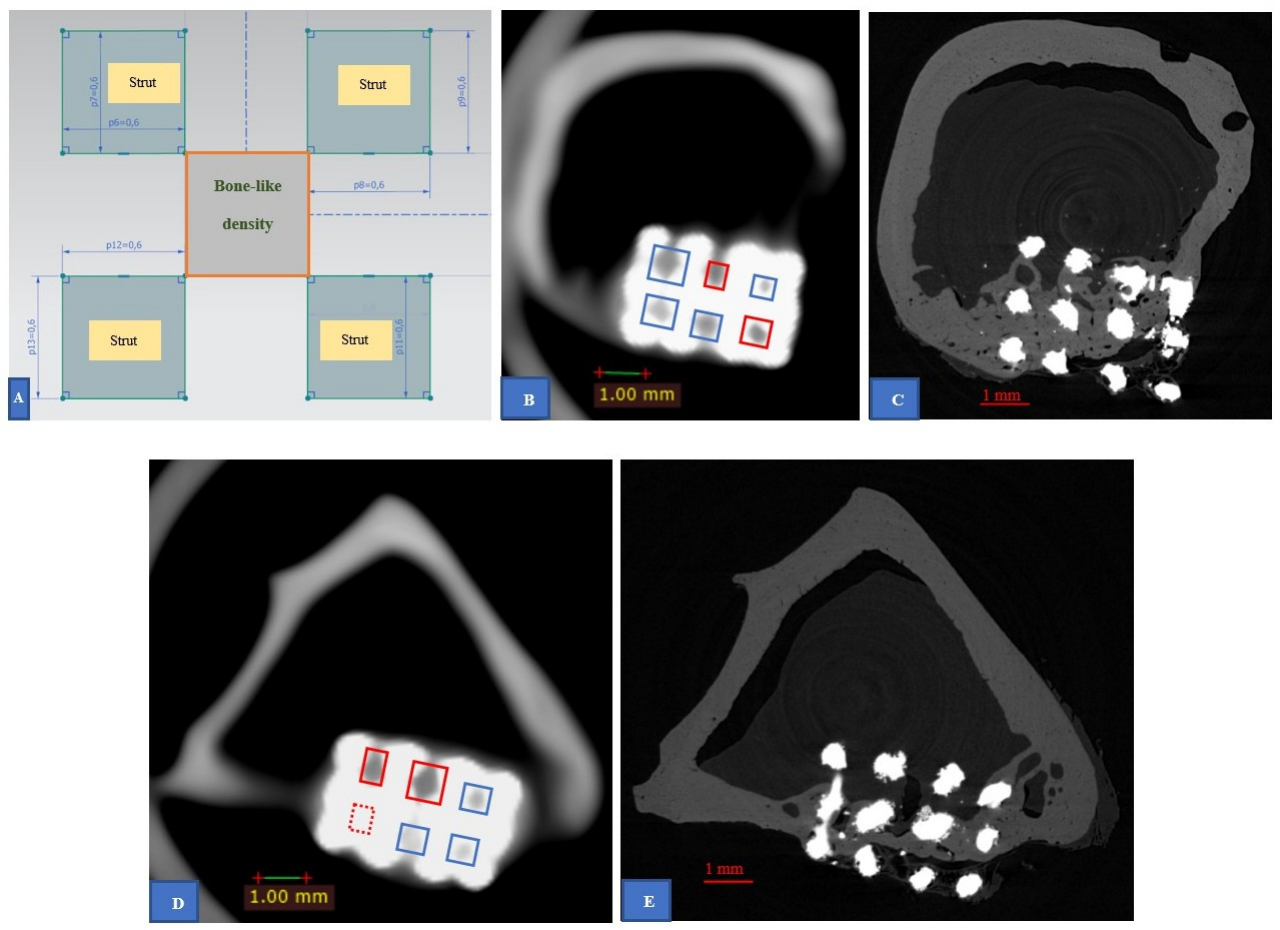
(color) Definition of the presentation of bone-like material inside a pore. (**A**) Cross section of a pore surrounded by four struts illustrates the area (marked by orange rectangle) that was used to evaluate the density. (**B**) Example of CBCT result (sample 10) illustrates the densities inside the blue rectangles that were considered having bone-like material whereas the densities inside the red rectangles were considered having no bone-like material. (**C**) The corresponding section of (**B)** obtained from μCT data. (**D**) An example of CBCT results (sample 8) that illustrates the bone-like density (blue rectangle) and no bone-like density (red rectangle). The red-dotted rectangle illustrates the pore considered having no bone-like density due to the artefact from surrounding metallic structure that made it unable to be evaluated. (**E**) The corresponding section of (**D)** obtained from μCT data.

High-resolution X-ray CT imaging of the four samples were performed at the Australian Synchrotron IMBL beamline with a Ruby detector at 5.9 μm pixel size and sample to detector distance of 225mm. For each sample, a total of 1800 X-ray projection images were acquired at a monochromatic beam energy 60keV. Flatfield and darkfield images have been acquired immediately before and after the projections for background correction prior to CT slice reconstruction. Phase-retrieval and ring-artefact correction algorithms were used to minimize artefacts in CT reconstruction. The CT reconstructed pixel values in the bright areas shown in Fig 5C and 5E were consistent with the known linear absorption coefficient (LAC) of the Ti6Al4V [26], which is about 2.95cm^-1^ at 60keV. The grey pixel values were associated with the bone while the dark-grey pixel values were associated with the soft tissues.

The observation of bone-like material filled in the pores was demonstrated in Fig 5. In this study, we considered the pores marked with red-dotted rectangles having no bone-like material filled in because the noise from metallic structures made the CBCT images difficult to observe and caused the contents in these pores difficult to evaluate (Fig 5D). The total of pores counted in the 4 implants scanned by μCT was 240. The pore that was considered to have bone-like material, we noted as “1”, otherwise we noted as “0”. The same analysis procedure was also carried out on CBCT data for 240 pores (S1–S3 Files) to evaluate its sensitivity, specificity, positive predicted value and negative predicted value.

### Statistical analyses

The rabbit’s weight before implantation, the time of explantation were set as independent variables. The number of pores which were observed to have bone ingrowth by CBCT (bone-contacting pores, non-bone-contacting pores and total pores) and the first date of hind legs weight bearing were chosen as the primary outcomes. The statistical analysis of the data was performed using Spearman’s correlation in SPSS Statistics 25 (IBM Inc., Chicago, IL, USA). Differences are considered significant at α = 0.05. In this study, μCT data were considered as the gold standard to evaluate the sensitivity, specificity, positive and negative predicted value of CBCT. The values were all rounded to zero decimal places.

## Results

### Overview

The mean weight of rabbits was 2.80 kg (Table 1). Particularly, the mean weights of rabbits obtained from group 1 and group 2 were 2.82 kg and 2.77 kg, respectively. The mean time of extraction was 22.67 weeks after surgery (Table 1). The postoperative rehabilitation of the rabbits progressed without any evidence of general and local complications such as infection, fracture of bone or implant. Generally, on postoperative day 7, all of the rabbits could stand on both their hind legs (Table 1). There was no correlation between the rabbits’ weight before implantation and the first postoperative date that the rabbits being able to stand up on both their hind legs (p = 0.466).

**Table 1:** Data records and CBCT scan analysis of 12 samples (N = 12).

Rabbit number	Weight before implantation (kg)	Time of explantation (weeks)	First postoperative date being able to stand up on both hind legs (days)	Total pores (*)	Bone-contacting pores (**)	Non-bone-contacting pores (***)
1	2.55	28	7	27	23	4
2	2.95	32	4	40	33	7
3	2.95	18	6	38	30	8
4	2.55	16	5	37	31	6
5	2.90	17	5	40	32	8
6	2.85	32	5	39	33	6
7	2.60	27	4	40	31	9
8	2.95	29	6	42	37	5
9	2.50	20	4	39	30	9
10	3.20	14	5	37	35	2
11	2.85	13	5	38	33	5
12	2.75	26	3	37	30	7

(*) Total pores: the number of pores having bone-like density assessed by CBCT.

(**) Bone-contacting pores: the number of bone-contacting pores having bone-like density assessed by CBCT.

(***) Non-bone-contacting pores: the number of non-bone-contacting pores having bone like density assessed by CBCT.

The prediction of the implant’s mechanical properties was carried out employing ANSYS finite element software. Solid tetrahedral elements SOLID187 were used in all analyses. The material properties of Ti6Al4V used in calculation include Young’s modulus of 107 GPa, Poisson ratio of 0.323 and a density of 4405 kg/m^3^. The results are illustrated in Table 2. Notably, in the longitudinal direction relating to axial loading force, the estimated equivalent Young’s modulus is 31.31 GPa whereas the prediction of compressive yield strength is 108.87 MPa (S4 File).

**Table 2:** Prediction of the implants’ mechanical properties using finite element analysis.

Direction	Equivalent Young’s modulus (GPa)	Compressive yield strength (MPa)	Notes
Z (Longitudinal)	31.31	108.87	See Fig 1 in S4 File for the orientation of forces
X (Transverse)	31.98	110.94	
Y (Transverse)	32.48	110.22	

### Macroscopic evaluation of the extracted samples

During the procedures for sample extraction, we noted that all of the exterior surface of implants that had had contact with the lateral muscle compartment were covered by membranous tissues (Fig 4D). There were three samples partially covered by bone tissue (S2 Fig).

### CT-scan evaluation

**Fig 6:**
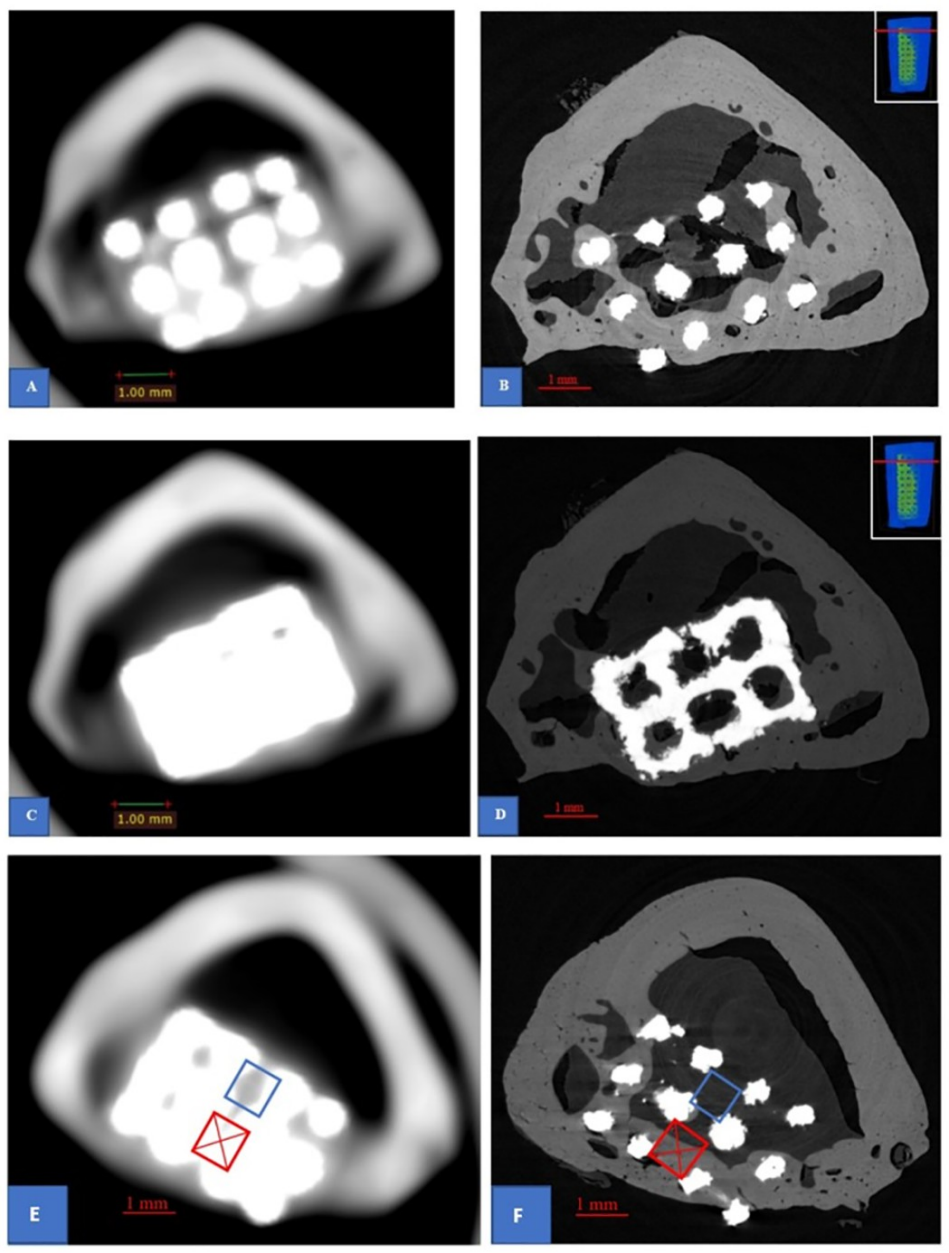
Comparison of CBCT and μCT data. CBCT sections (**A**, **C**) and corresponding μCT sections (**B**, **D**) obtained from sample 9. (**A-B**) The slices at the level of pores. (**C-D**) The slices at the level of horizontal struts, leading to metallic artefact in cone beam image. The top right corner images of (**B)** and (**D)** indicate the level of the slices in the specimens. The illustration of over-evaluation of CBCT (**E**) with bone-like density presented in non-bone-contacting pore (blue rectangle) and corresponding evaluation of μCT (**F**) showed no bone-like density in the same pore. The under-evaluation of CBCT can be caused by the low resolution or metal artefact leading to the difficult assessment of bone-like density in the pore (red rectangle with cross) when comparing to the μCT data.

In all specimens, CBCT showed that the tissues having bone-like density was presented in both bone-contacting pores and non-bone-contacting pores (Fig 6A). Further evaluation with μCT supports this finding (Fig 6B and 6D). There was no correlation found between the explantation time and the number of pores (total pores, bone-contacting pores, non-bone-contacting pores) in each implant having bone-like density (p = 0.188, p = 0.956, p = 0.718, respectively). μCT was considered as a gold standard to evaluate the bone ingrowth into the pores. The under- and over-evaluation of bone ingrowth into the pore was demonstrated in Fig 6. CBCT has a positive predictive value for bone-like density presented in the pores of implants (the total of 240 pores, 60 pores per implant) of approximately 93%. Its negative predictive value for the pores that did not present bone-like density is approximately 70%. Its sensitivity and specificity were 85% and 84%, respectively. Its type I error rate (false positive) and type II error rate (false negative) were 16% and 15%, respectively. In all specimens, no stress shielding effect was observed in both CBCT and μCT data.

### Histological examination

**Fig 7:**
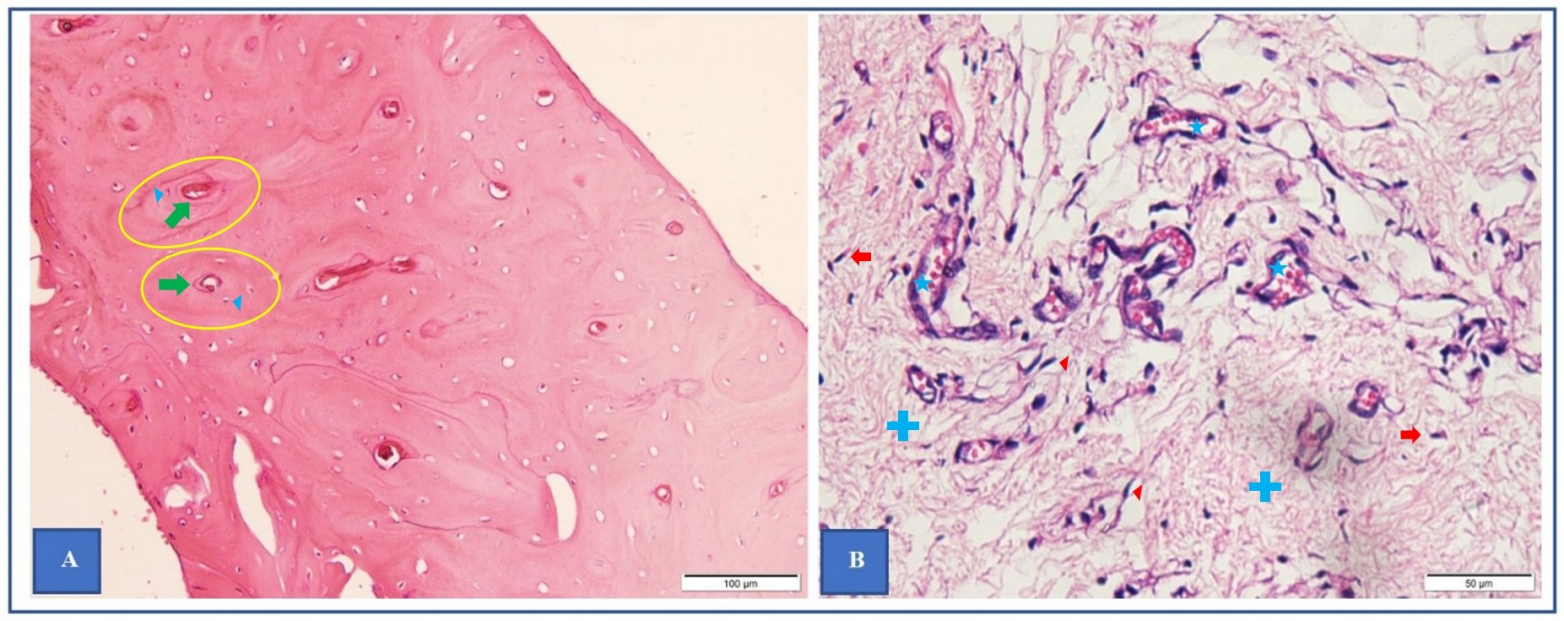
(color) Histological evaluation of specimens. Histological examinations of bone-like tissue (**A**) and membranous tissue (**B**) obtained from sample 2 with hematoxylin and eosin stainning. (**A**) Specimen composed of mature bone tissue with lamellar arrangement (yellow circles) surrounding Haversian canals (green arrows) and osteocytes (blue arrow heads). (**B**) Specimen composed of high-vascularized collagenous tissue (blue crosses) with fibroblasts (red arrow heads), fibrocytes (red arrows) and blood vessels (blue stars).

Histologically, all pieces of bone-like tissue collected from the non-bone-contacting pores of 12 specimens were mature bone with lamellar arrangement surrounding Haversian canals (Fig 7A). In terms of the membranous samples, the microscopic analyses also showed that they were composed of high-vascularized collagenous tissue with fibroblasts, fibrocytes and blood vessels (Fig 7B). And no malignant cell was observed in all specimens.

## Discussion

In this study the implant structure has an estimated equivalent Young’s modulus of 31.31 GPa and equivalent compressive yield strength of 108.87 MPa employing finite element analyses. According to the Gibson—Ashby model [27] for estimating the Young’s modulus (Eq 1), the predicted value is 26.75 GPa.

$$E_{Eff}=E_{s}*{\left(q_{rel}\right)}^{2}$$ (1)

Where *E*_*Eff*_ is the effective modulus of elasticity of porous component, *E*_*s*_ is the modulus of elasticity of the solid material and *q*_*rel*_ is the relative density of the porous component [28].

The above predicted moduli are relatively comparable to the Young’s modulus of cortical bone of human [7, 8, 29] and rabbit [30], ranging from 18 to 29 GPa and higher than those of the implants used in previous studies [14, 31, 32] mainly due to the lower porosity. In particular, Taniguchi et al. [14] used a diamond lattice structure having porosity between 62 to 66 per cent to achieve the Young’s modulus ranging from 557.4 MPa to 661.4 MPa for implantation to the metaphyseal bone of rabbits. The zone is generally made up of trabecular bone with elastic modulus ranging from 0.5 GPa to 10 GPa [29, 33]. It should be noted that the predicted compressive yield strength of the lattice structure used in our study has the value of 108.87 MPa, which is comparable to that of human cortical bone, ranging from 100 to 150 MPa [33].

Histological examination showed that the cortical bone ingrowth into non-bone-contacting pores reached maturity after 13 weeks. This finding is in broad agreements with the previous studies [14, 22, 31, 32, 34] about the potential of porous Ti6Al4V scaffolds with pore size of around 600 μm to facilitate osseointegration in bone defect. Our study has supplemented the study of Jan Wielding et al [21] with both radiological examination using high resolution CT-scan (100 μm and 5.9 μm effective imaging pixels in CBCT and μCT, respectively) and histological assessment of new bone ingrowth’s quality. Furthermore, our study also found that the membranous tissues formed at the exterior side of implants might be relatively comparable to the Masquelet induced membrane [35, 36]—a high vascularized tissue to facilitate bone healing and that no malignant cell was observed in all histological specimens, including four specimens (two specimens of bone-like tissue and two specimens of membranous tissue) extracted at 32 weeks after implantation.

**Fig 8:**
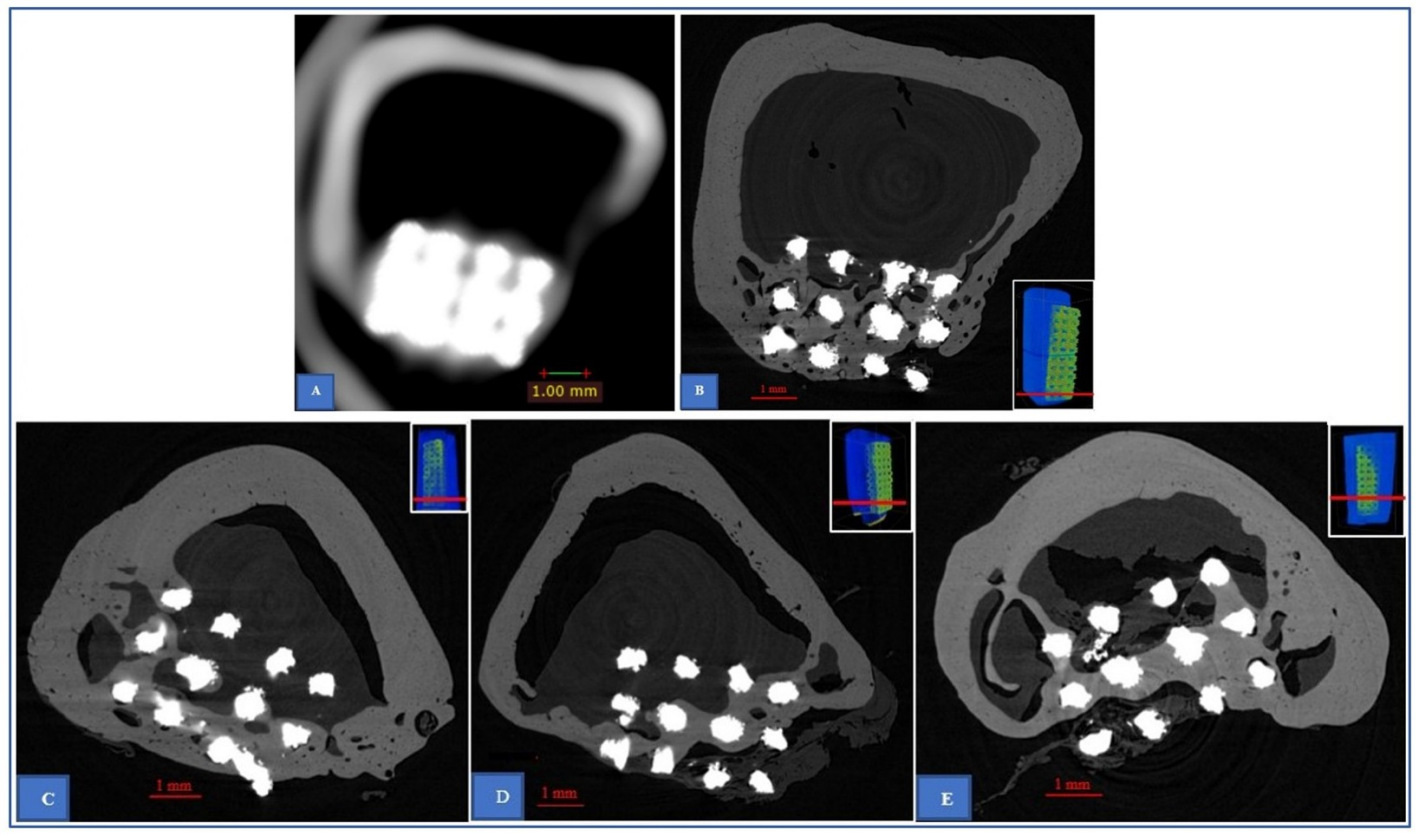
Bone ingrowth into pores at different time points by μCT and CBCT assessments. The CT-scan images obtained from samples 10 (**A**-**B**), sample 9 (**C**), sample 8 (**D**) and sample 6 (**E**). The small images with red lines indicate the level of the slices in the specimens. Regarding the bone ingrowth into the pores, the two samples from the first group (**A**-**B**: 14 weeks; **C**: 20 weeks) have higher porosity than the two samples from the second group (**D**: 29 weeks, **E**: 32 weeks).

Regarding CT-scan assessment, as illustrated in Fig 7, μCT revealed more detailed information than CBCT did to evaluate more accurately the bone ingrowth into the porous implants. By examination of the 4 samples’ datasets, our study found that the positive predictive value of CBCT for the presentation of bone-like density in the pores is 93 percent, its negative predictive value for having no bone-like density is 70 percent and that its sensitivity and specificity were 85 and 84 percent, respectively. This finding is in good agreement with the results reported by Van Dessel et al [37] when they investigated the structural pattern of the alveolar bone using different CBCT system. They [37] found that the ProMax 3D Max, NewTom GIANO, Cranex 3D, 3D Accuitomo 170 and Carestream 9300 demonstrated CBCT machines can achieve the level of accuracy and reliability approaching μCT. However, their study [37] mainly focused on the morphometric of trabecular bone. To our knowledge, our study is the first attempt to compare the results obtained from clinical CBCT and μCT in evaluating the cortical bone ingrowth into porous implants. Despite its low resolution when comparing to μCT, CBCT is a promising system to evaluate the bone ingrowth into a porous titanium alloy implant while it is still impossible to perform μCT in living human body. Nevertheless, care should be taken when analysing CBCT data due to its low-resolution images, even though its image resolution is still higher than conventional CT-scan. For example, Fig 8A shows that the CBCT scan data give impression of good bone healing but in fact, it provides inadequate detail of the quality of bone ingrowth into the pores. The corresponding section obtained from μCT (Fig 8B) show that this bone ingrowth still has high porosity. Although the Wolff’s law states that bone is continuously remodeling under the influence of loading [20], the high porosity of bone could be unsafe for the patient who prefers full weight bearing in the early clinical setting. Fig 8 also provides the comparison of bone ingrowth quality at different times of explantation. In our study, the porosity was observed to reduce over time. This finding suggests that the longer the porous device is implanted in the bone, the less porosity of the bone ingrowth may result. It also supports the Wolff’s law that the remodeling process is guided by mechanical forces. However, due to the small number of specimens, we only investigated one porous structure having the intended pore size of 600 μm which was demonstrated to have high ability to induce bone ingrowth into the pore in previous studies [14, 31, 38, 39]. Further studies are needed to find the optimum pore size for cortical bone ingrowth.

## Conclusion

Ti6Al4V porous implants fabricated by EBM technology were used to replace the diaphyseal tibial bone defect in 12 New Zealand white rabbits to investigate the ability of cortical bone ingrowth into the Ti6Al4V scaffolds. It was found that

1. both the CT-scan and histological results showed that cortical bone ingrowth into the porous implants,
2. fully porous titanium alloy implants have the potential for reconstruction of diaphyseal bone defect due to its good ability of osseointegration,
3. CBCT is a promising method for evaluating the bone ingrowth into the porous implants, especially for those used in living human, and
4. finally, further studies are needed to find the optimum pore size for cortical bone ingrowth.

## Supporting information

S1 Fig The illustration of porous implant after replacing the tibial defect with press-fit method. (TIF)

S2 Fig The illustration of specimen from sample 9 (view from lateral to medial). The bone tissue partially covered the implant (A) and its corresponding axial slice obtained from μCT data (B). The green line in (A) indicates the corresponding level of slice in (B).
(TIF)

S1 File CBCT analysis. (XLSX)

S2 File MicroCT analysis. (XLSX)

S3 File CBCT—MicroCT comparison. (XLSX)

S4 File Finite element analyses of implant’s mechanical properties. (DOCX)

S5 File The ARRIVE guidelines checklist. (DOCX)
