# Supplementary figures and images for: Osseointegrability of 3D-printed porous titanium alloy implant on tibial shaft bone defect in rabbit model
Source: PLoS One. 2023 Sep 8;18(9):e0282457. doi: 10.1371/journal.pone.0282457 (PMC10490944; doi:10.1371/journal.pone.0282457)

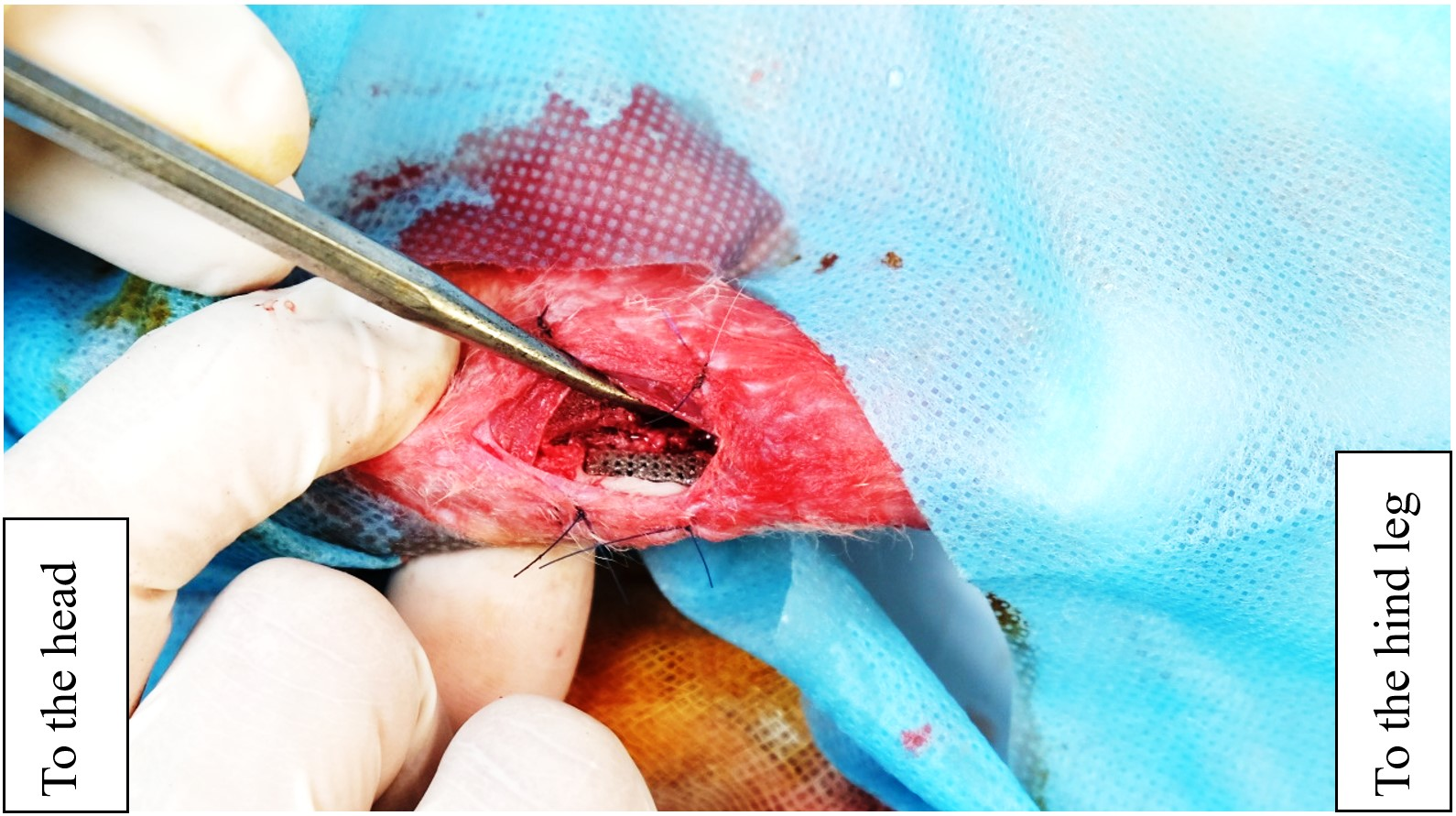

Supplement: S1 Fig — (TIF) [file pone.0282457.s001.tif]

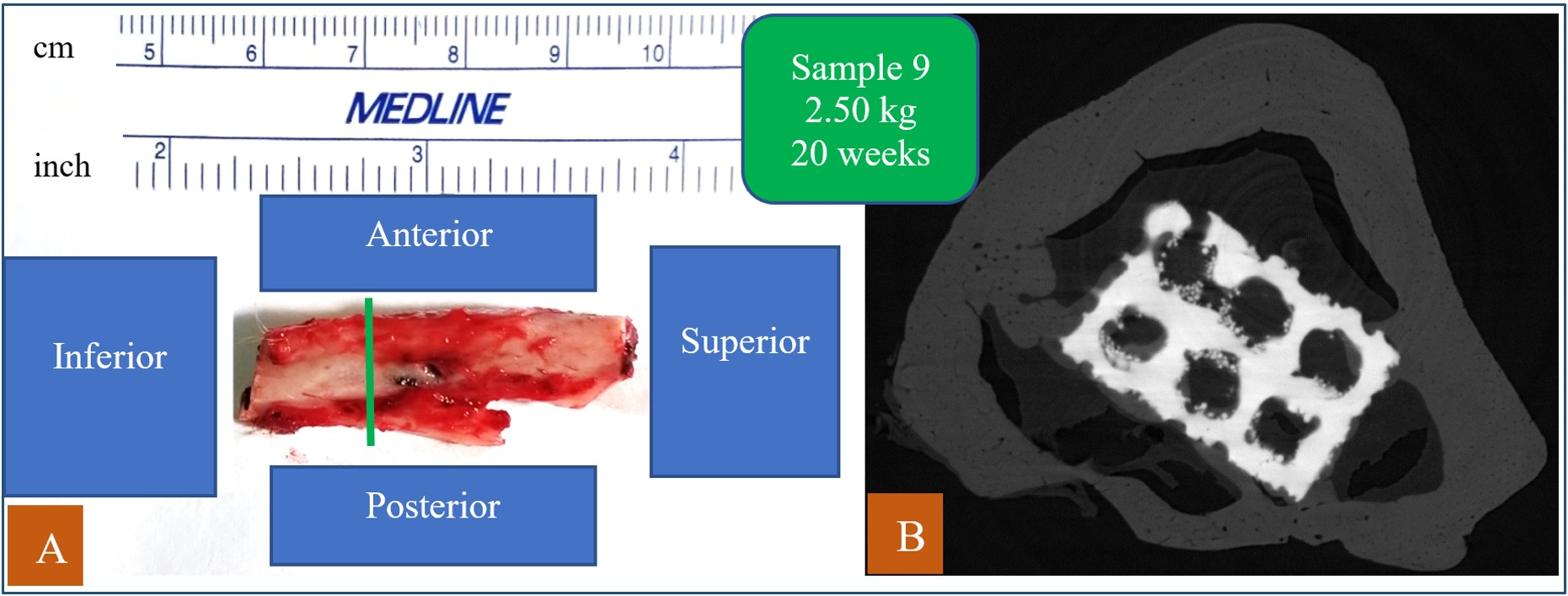

Supplement: S2 Fig — The bone tissue partially covered the implant (A) and its corresponding axial slice obtained from μCT data (B). The green line in (A) indicates the corresponding level of slice in (B). (TIF) [file pone.0282457.s002.tif]
