# Supplementary material for: Osseointegrability of 3D-printed porous titanium alloy implant on tibial shaft bone defect in rabbit model
Source: PLoS One. 2023 Sep 8;18(9):e0282457. doi: 10.1371/journal.pone.0282457 (PMC10490944; doi:10.1371/journal.pone.0282457)
Supplement: S4 File — (DOCX) [file pone.0282457.s006.docx]

**APPENDIX A**

**Finite element analyses**

We used ANSYS software (ANSYS, USA) for finite element analyses our implant’s mechanical properties, including the elastic modulus and the equivalent compressive yield’s strength.

For calculating the implant’s elastic modulus, we imported the STL model to ANSYS Mechanical software. The mechanical properties of Ti6Al4V used in calculation include: Young’s modulus 107 GPa, Poisson ratio 0.323, and a density 4405 kg/m^3^. The Static Structural module was used. Nonlinear Effects and Thermal Strain Effects were disable. Solid tetrahedral elements SOLID187 were used in the analysis (Figure 1). We assumed our porous implant as the solid one with the dimension of 2.4 (mm) x 3.6 (mm) x 12 (mm). Therefore, the equivalent surface area of its longitudinal axis was 2.4 (mm) x 3.6 (mm) = 8.64 x 10^-6^ m^2^.


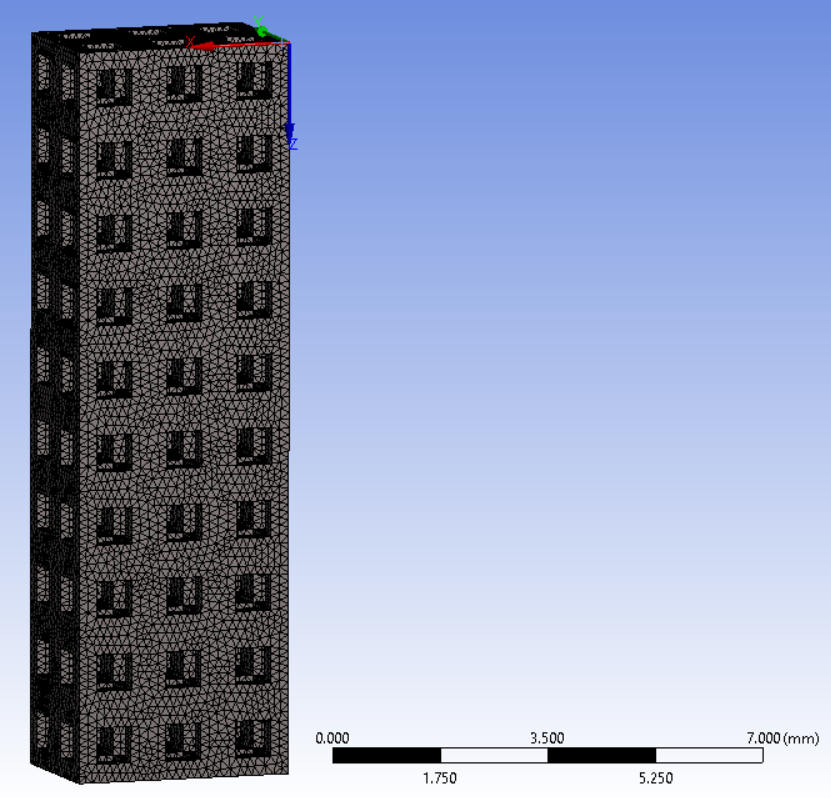


Figure 1: The tetrahedral mesh of the implant was used for the analysis.

The fixed support and displacement were configured as Figure 2. The displacement was set to 0.1 mm (ΔL). In our test, the Probe Tool was used to evaluate the Force Reaction at the surface received the displacement force (Figure 3).


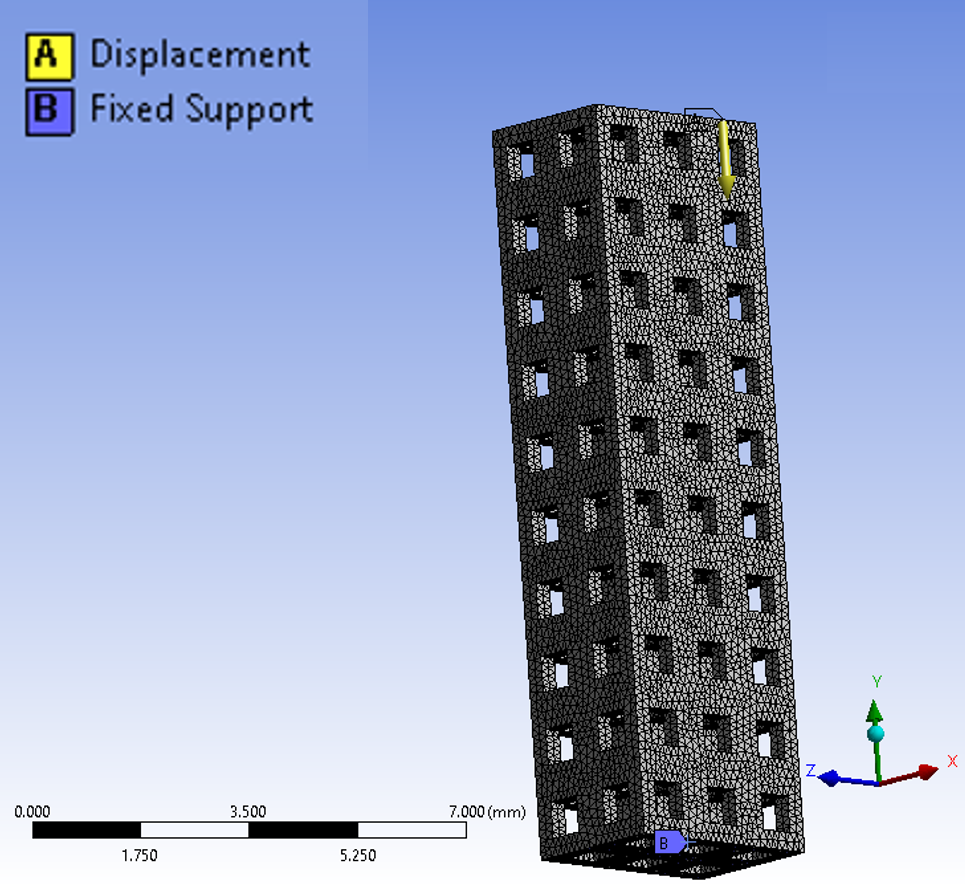


Figure 2 The Displacement vector (yellow arrow) of 0.1mm and Fixed Support (Surface B) was set for estimating the Young’s modulus of longitudinal direction (Z axis).


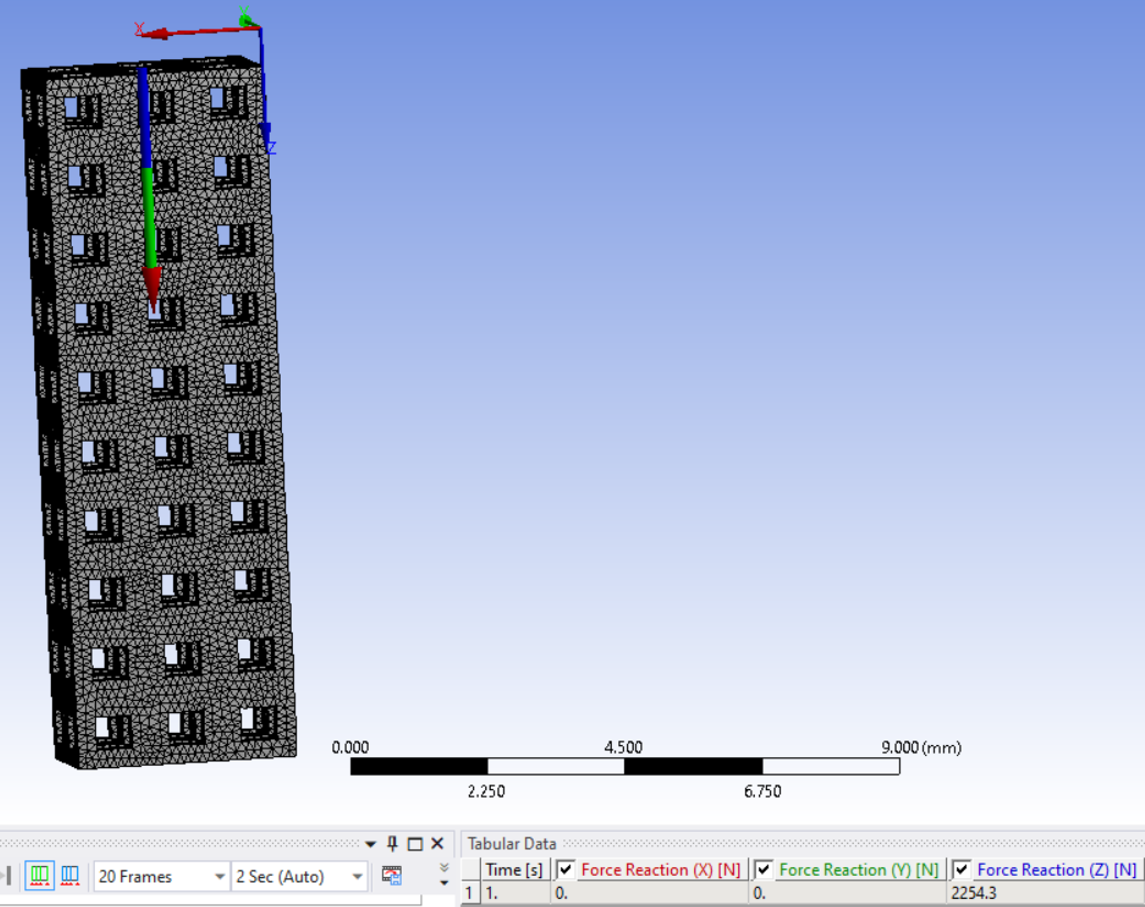


Figure 3 The Force Reaction at the displacement site was calculated.

By the analysis, the force reaction was 2254.3 N. As the linear behavior of the material, the estimated Young’s modulus was calculated by:

$Estimated Young^{'}s modulus=\frac{\mathrm{Stress}}{\mathrm{Strain}}$ =$\frac{\frac{\mathrm{Load}}{Surface area}}{\frac{L}{L}}$ = $\frac{\frac{2254.3}{8.64 x {10}^{-6}}}{\frac{0.1 x {10}^{-3}}{12 x {10}^{-3}}}$ = 31.3 GPa


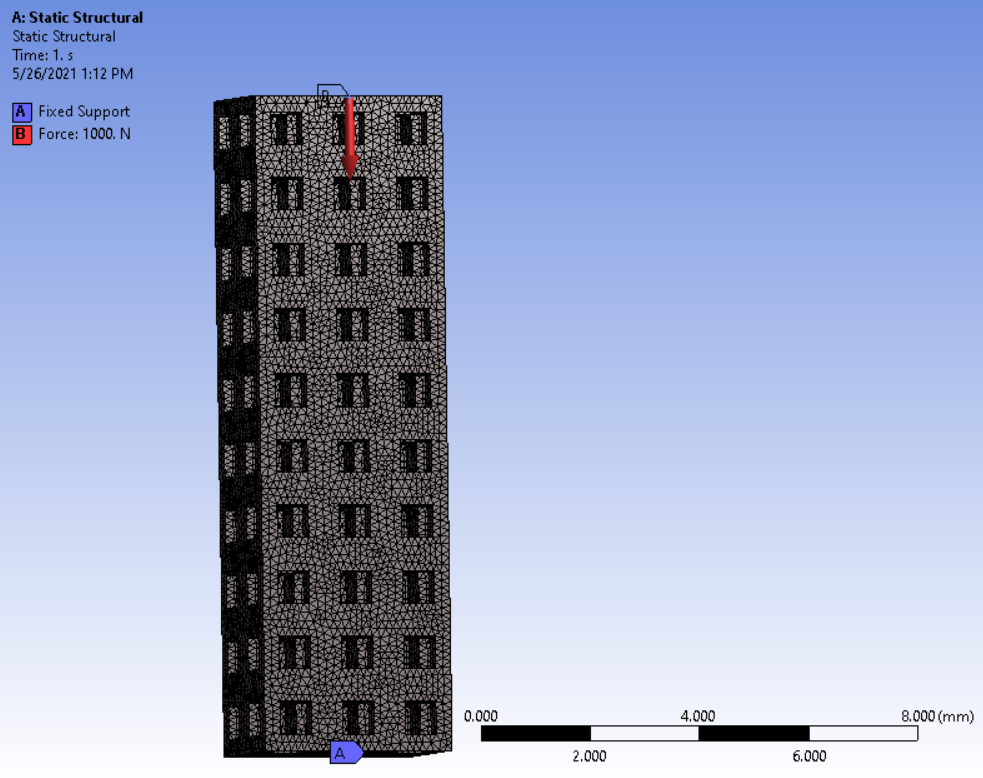
For estimating the Yield strength of the implant, the Force and Fixed Support was configured as Figure 4. The Force was set to 1000 N. The Maximal von-Mises stress was calculated, and this value was used to get the predicted Yield stress (Yield strength).

Figure 4 The Force vector (red arrow) and Fix Support (Surface A) was set for the analysis of equivalent compressive Yield strength.

$Equivalent Compressive Stress=\frac{\mathrm{Load}}{Surface area}$ $=\frac{1000}{8.64x{10}^{-6}}$= 115.7 MPa

With the equivalent compressive stress of 115.7 MPa, the Maximal von-Mises stress of the implant is 1034.4 MPa (calculated by Finite Element Analysis, Figure 5).


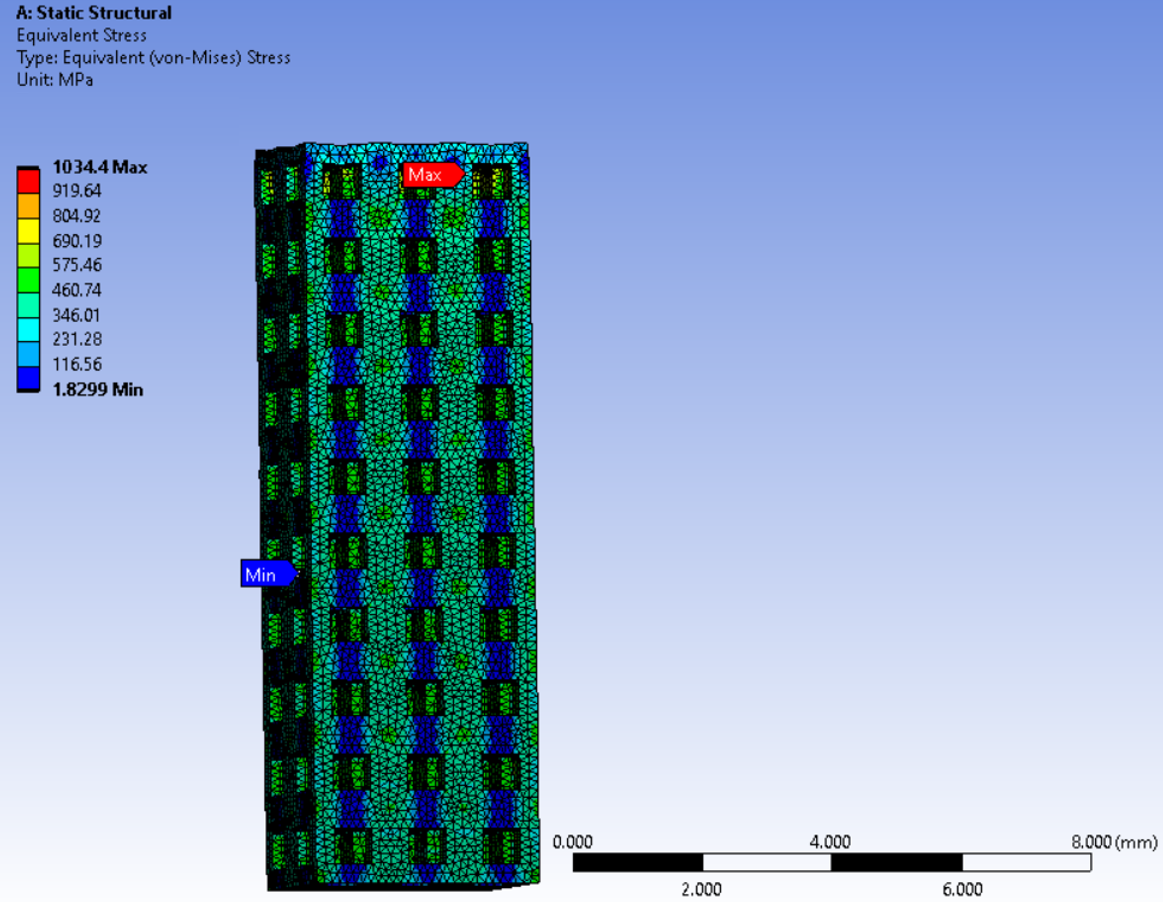


Figure 5 The final result of equivalent (von-Mises) stress analysis with the maximal value of 1034.4 MPa.

As the reported Yield strength of the as-built Ti6Al4V produced by EBM is 973 MPa [1], we estimated the Equivalent Yield Stress of our implant is 108.87 MPa as the following proportion:

$$\frac{Equivalent Stress: 115.7 MPa}{Equivalent Yield Stress: ? MPa}= \frac{Maximal von-Mises stress: 1034.4 MPa}{Yield Strength: 973 MPa}$$

We repeated the same steps for calculating Estimated Young’s modulus and Equivalent compressive Yield stress in other directions of loading boundary (Figure 6 – 13). The summary of the analyses was illustrated in Table 1.


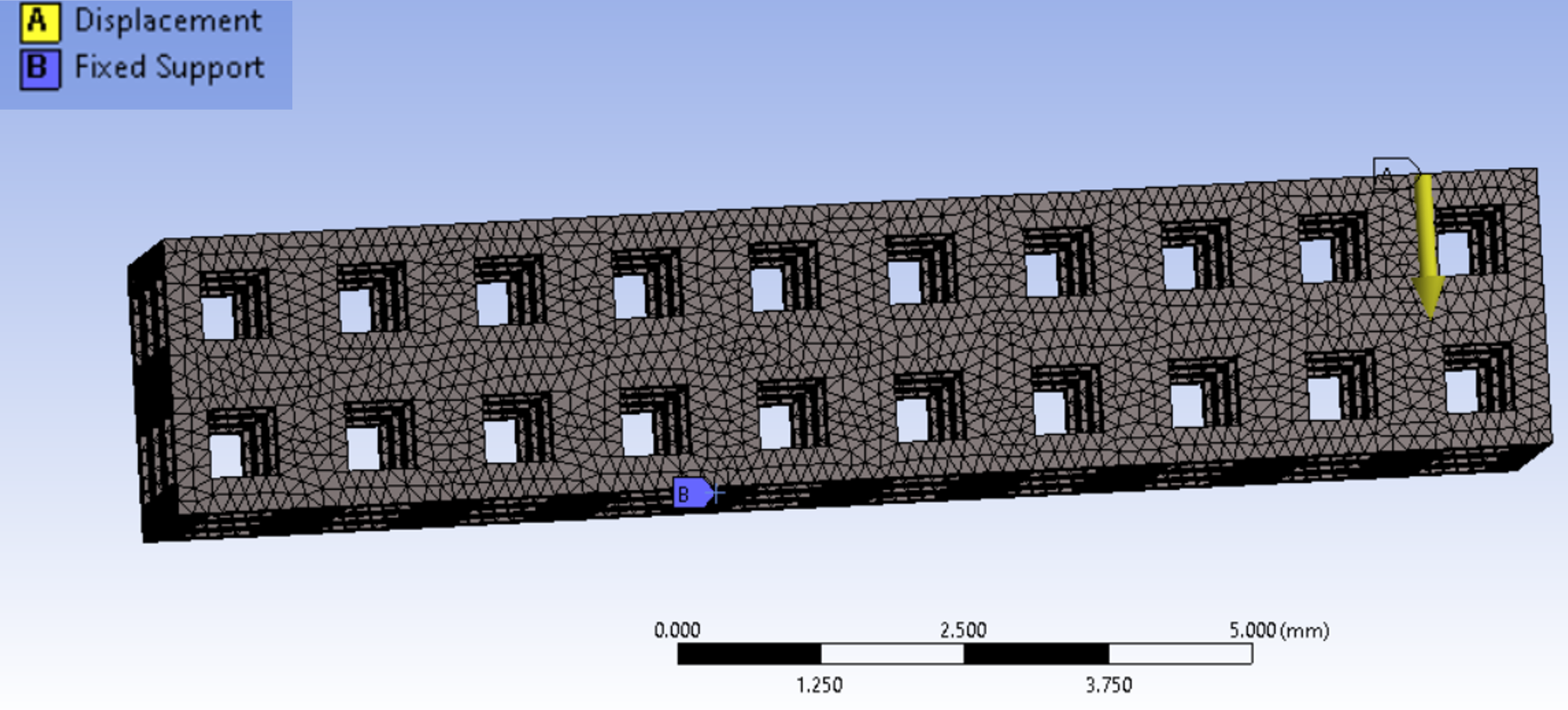


Figure 6 The Displacement vector (yellow arrow) and Fixed Support (Surface B) was set for estimating the Young’s modulus of transverse direction (Y axis).


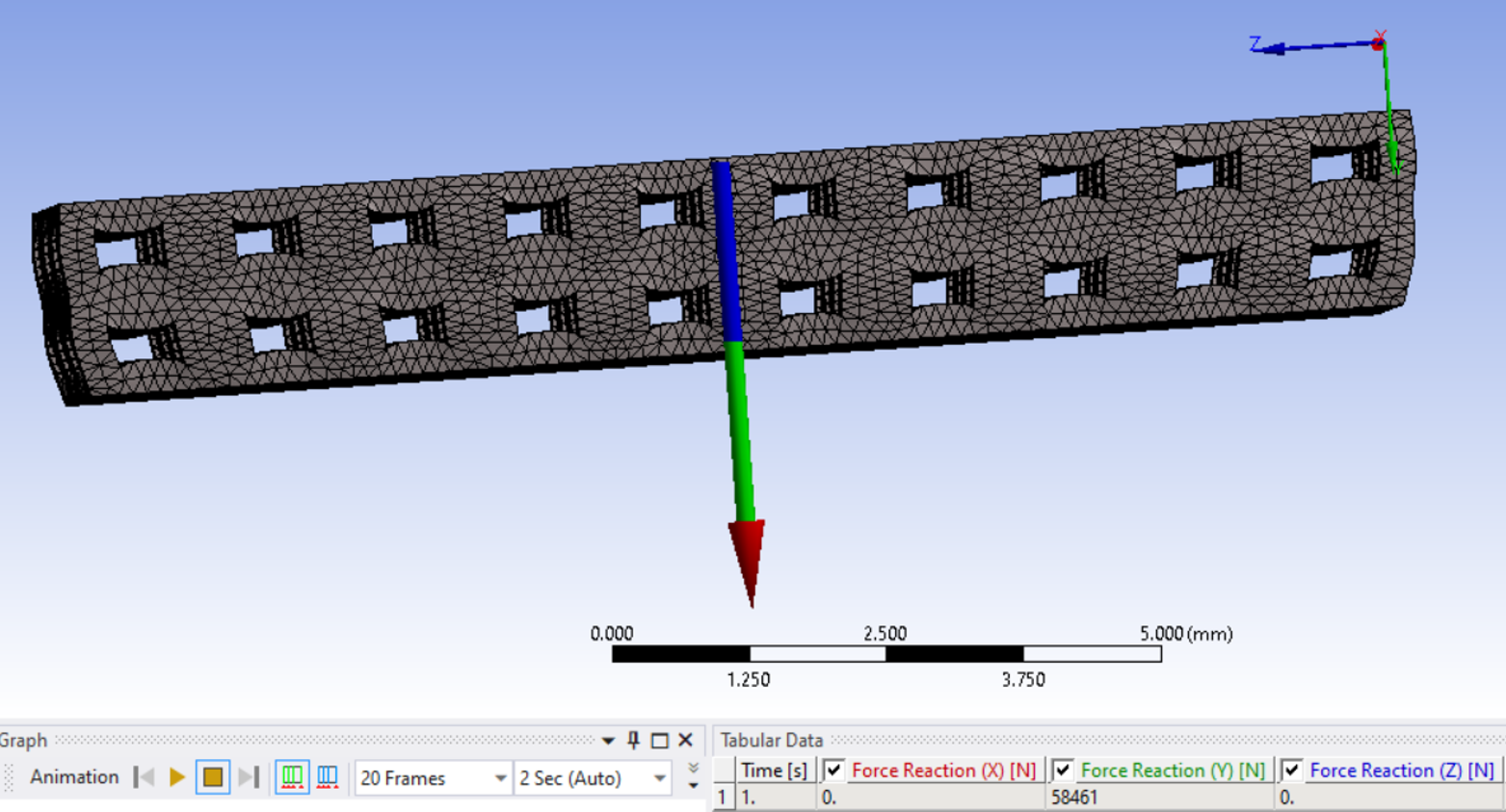


Figure 7 The Force Reaction at the displacement site was calculated.


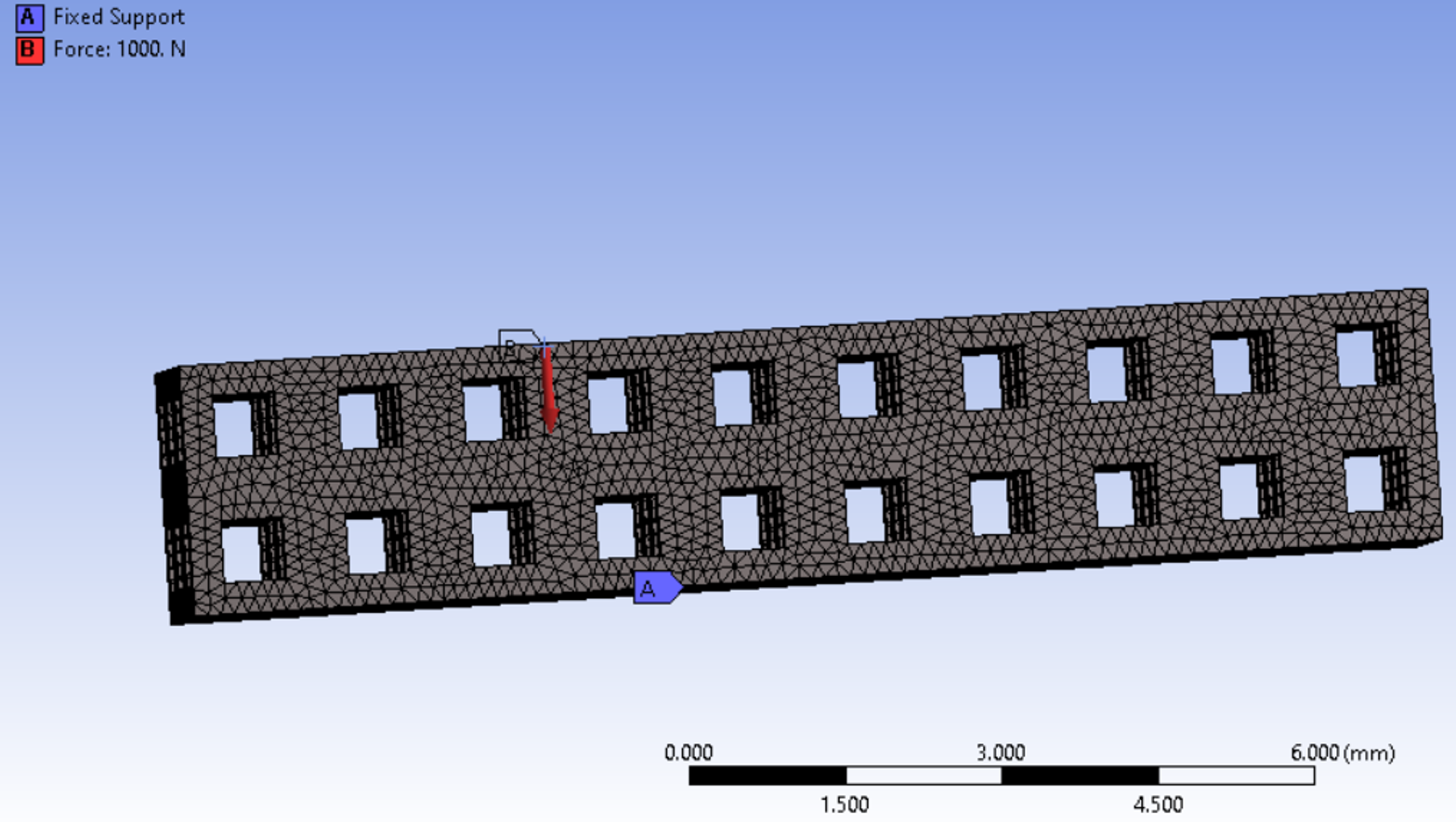


Figure 8 The Force vector (red arrow) and Fix Support (Surface A) was set for the analysis of equivalent compressive Yield strength.


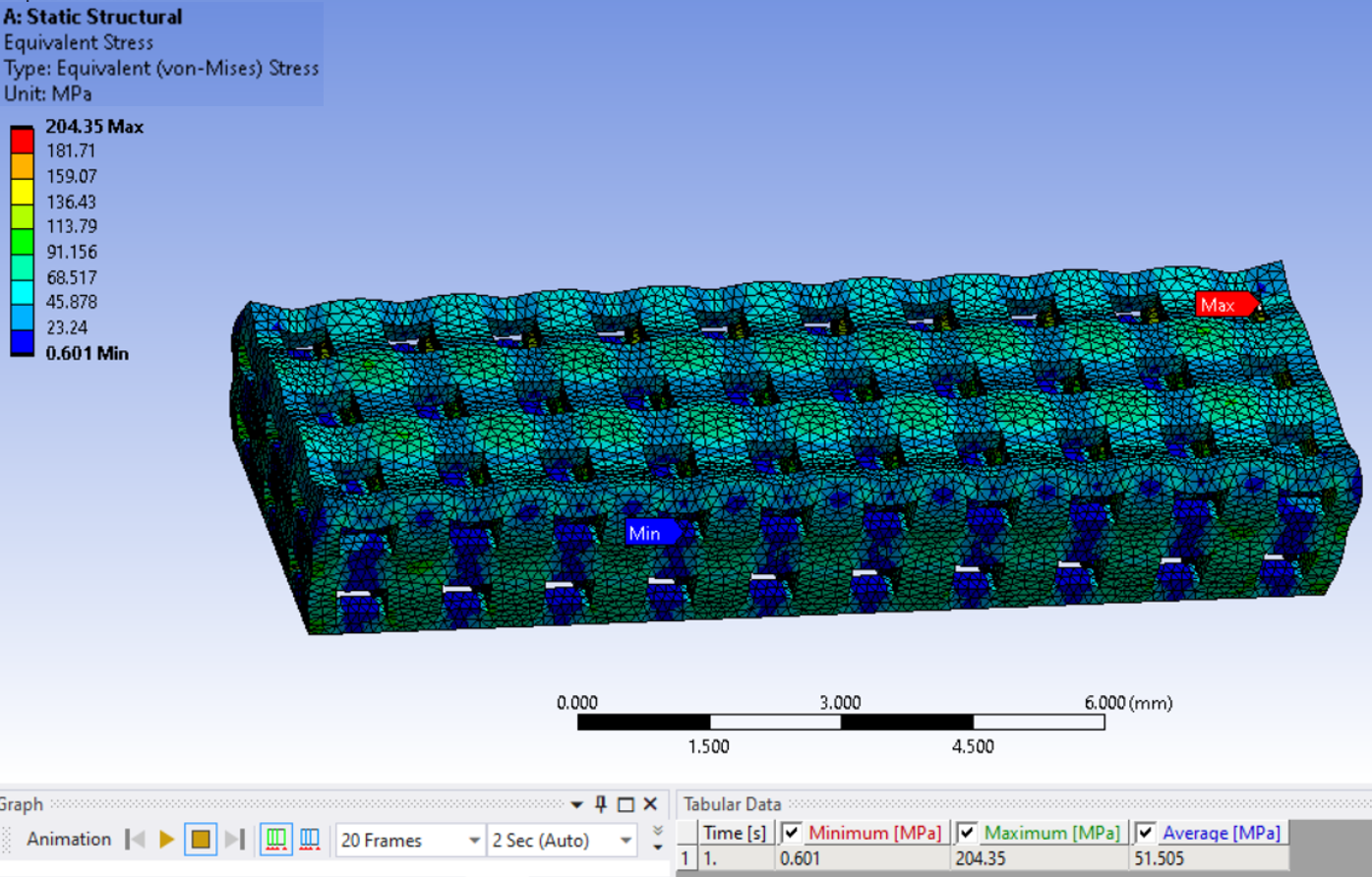


Figure 9 The final result of equivalent (von-Mises) stress analysis with the maximal value of 204.35 MPa.


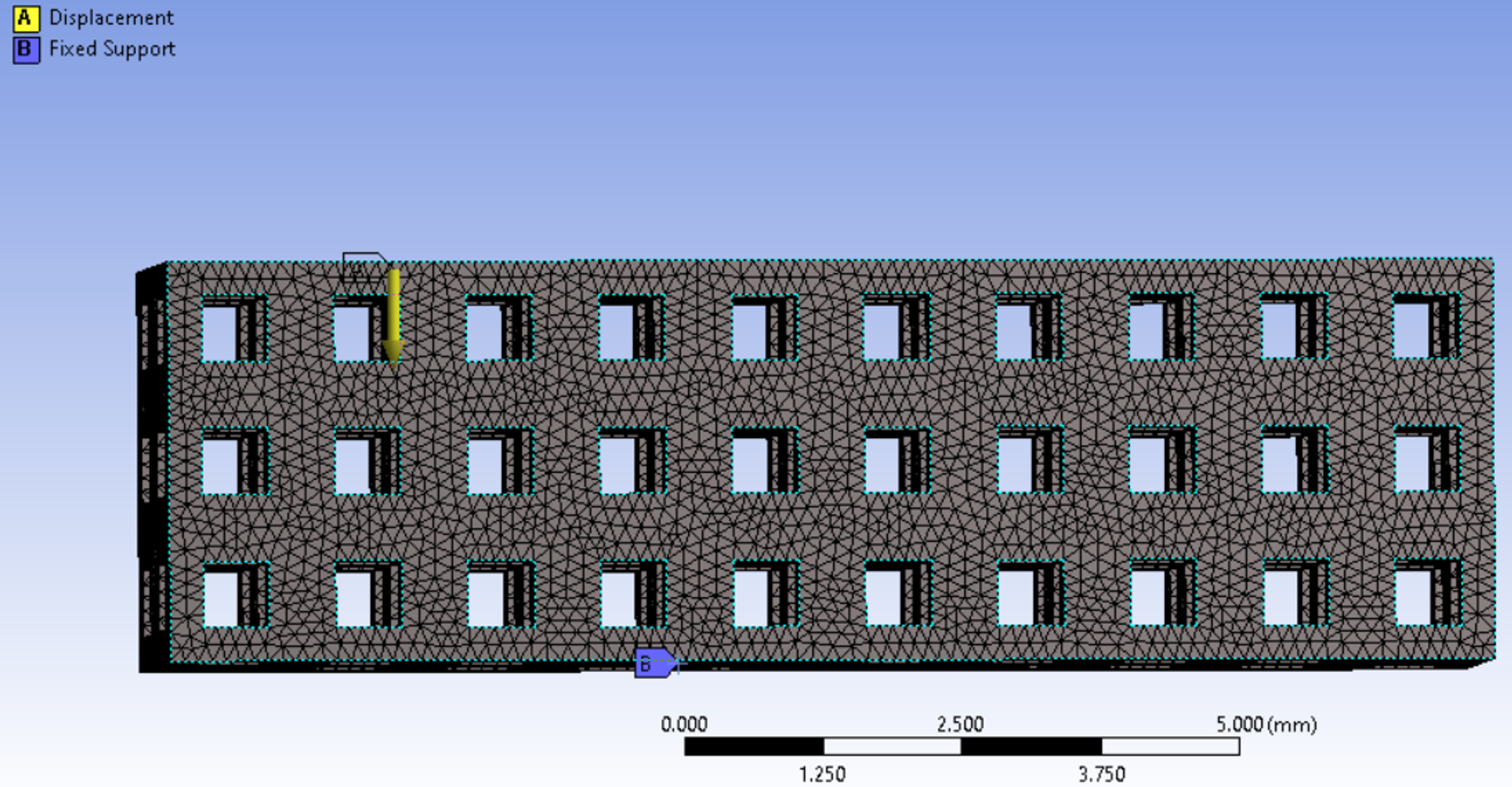


Figure 10 The Displacement vector (yellow arrow) and Fixed Support (Surface B) was set for estimating the Young’s modulus of transverse direction (X axis).


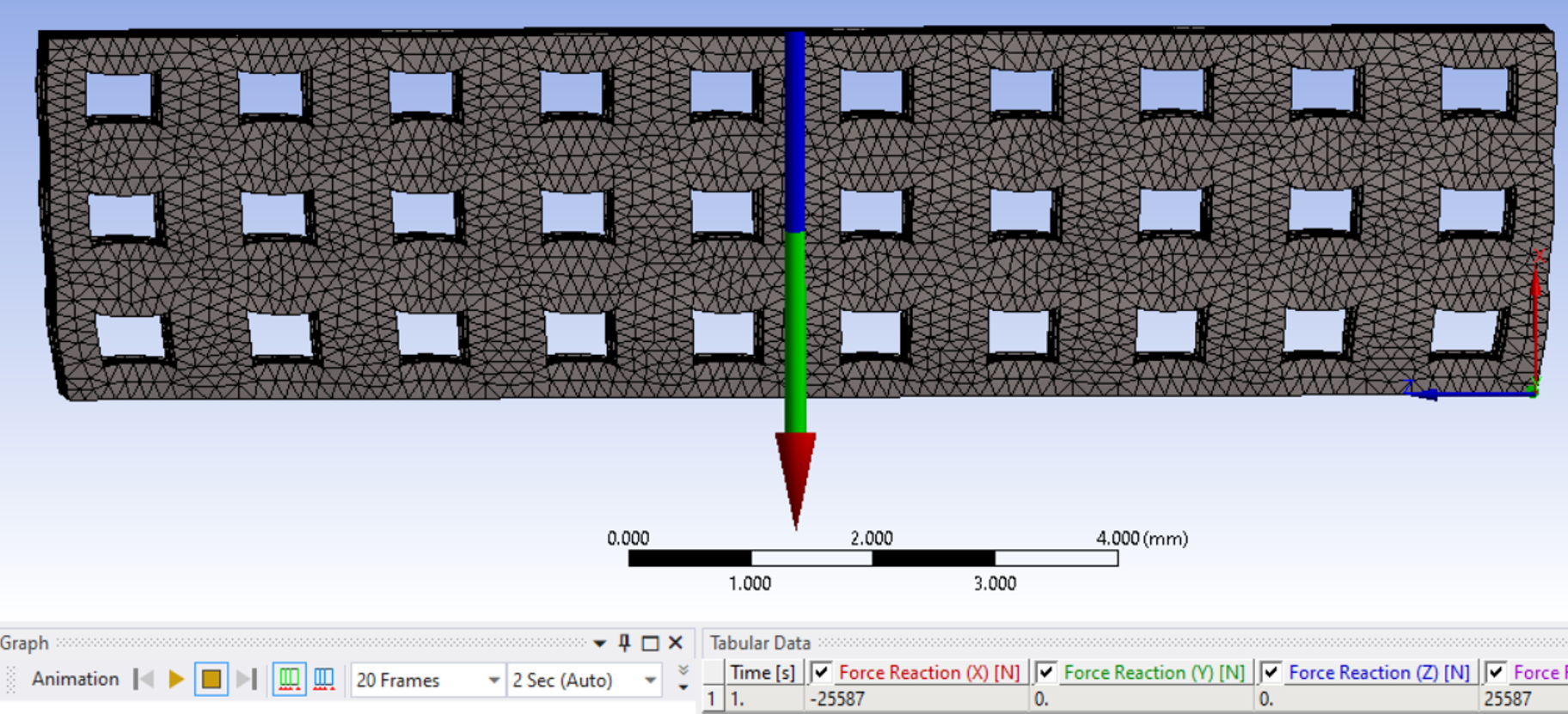


Figure 11 The Force Reaction at the displacement site was calculated.


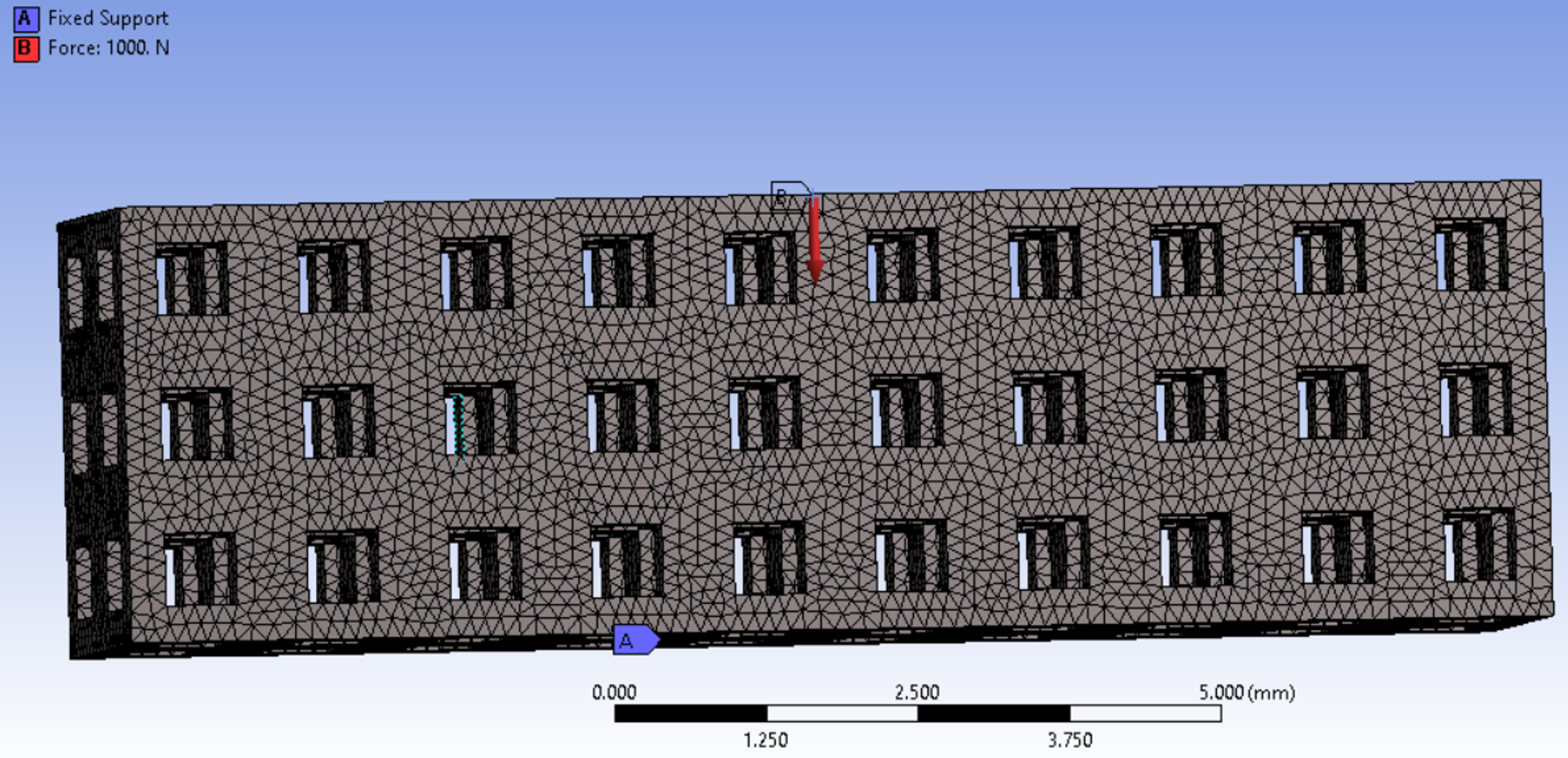


Figure 12 The Force vector (red arrow) and Fix Support (Surface A) was set for the analysis of equivalent compressive Yield strength.


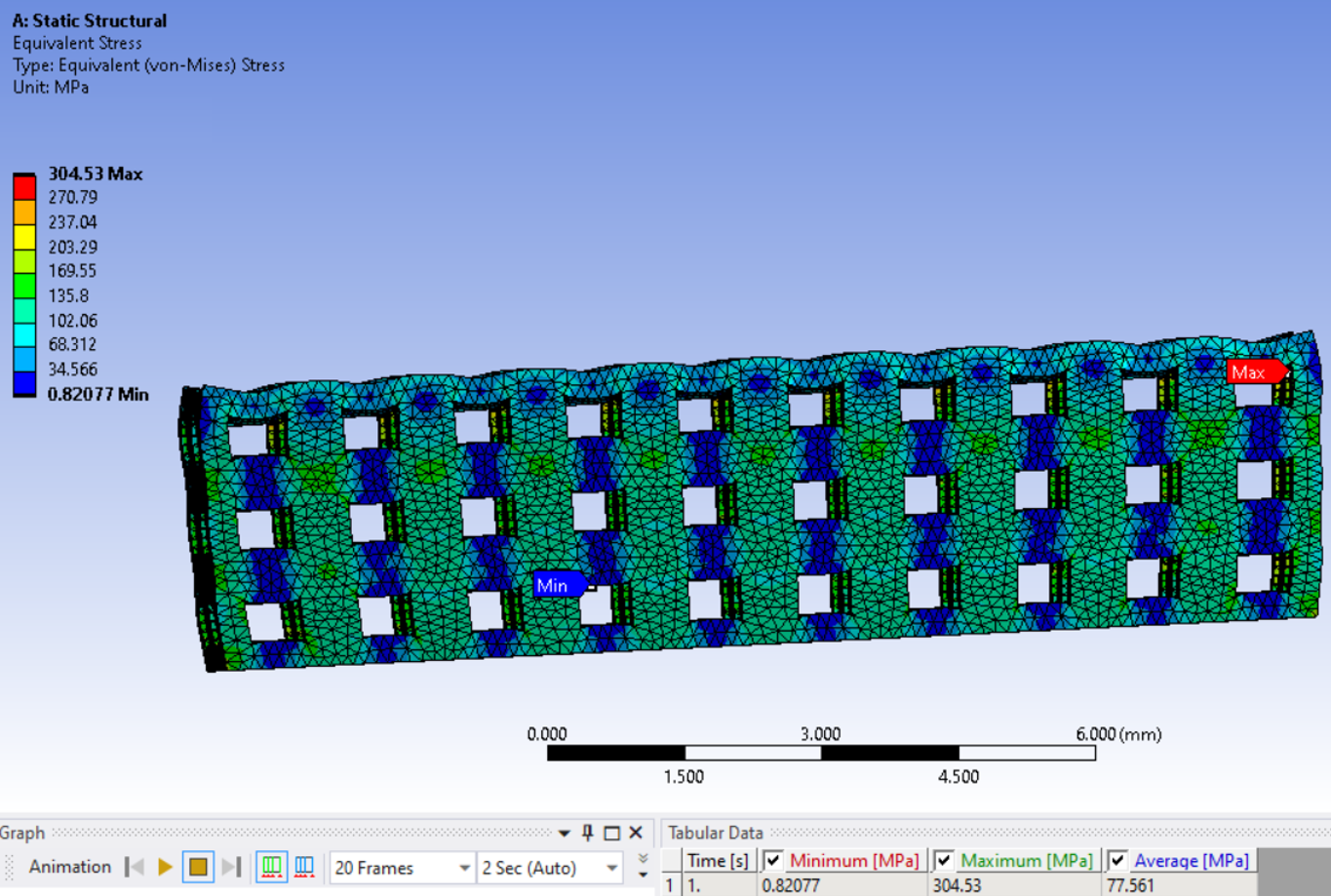


Figure 13 The final result of equivalent (von-Mises) stress analysis with the maximal value of 304.53 MPa.

Table 1 Summary of the results from finite element analyses

| **Direction** | **Equivalent Young’s modulus**  **(GPa)** | **Compressive yield strength**  **(MPa)** | **Illustration** |
| --- | --- | --- | --- |
| Z  (Longitudinal) | 31.31 | 108.87 | 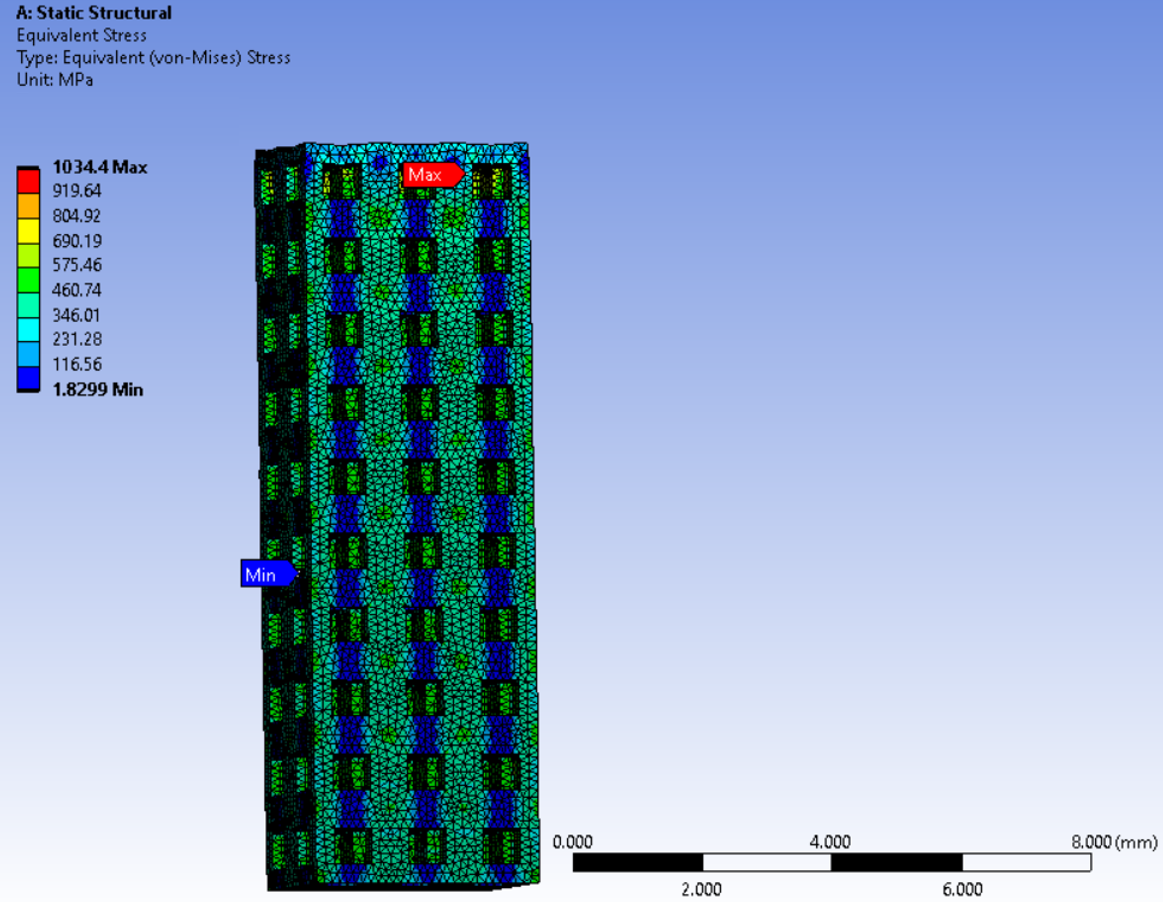 |
| X  (Transverse) | 31.98 | 110.94 | 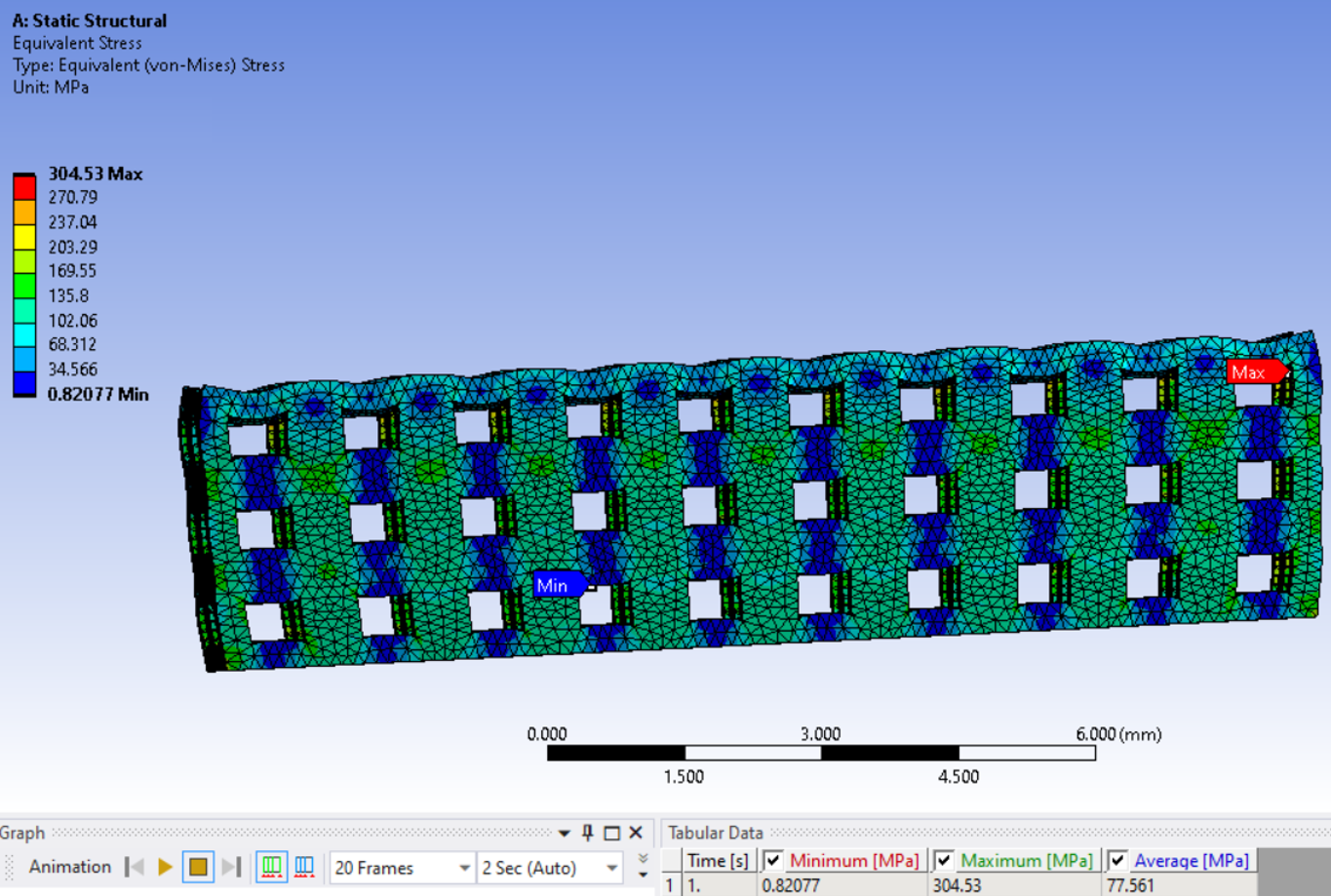 |
| Y  (Transverse) | 32.48 | 110.22 | 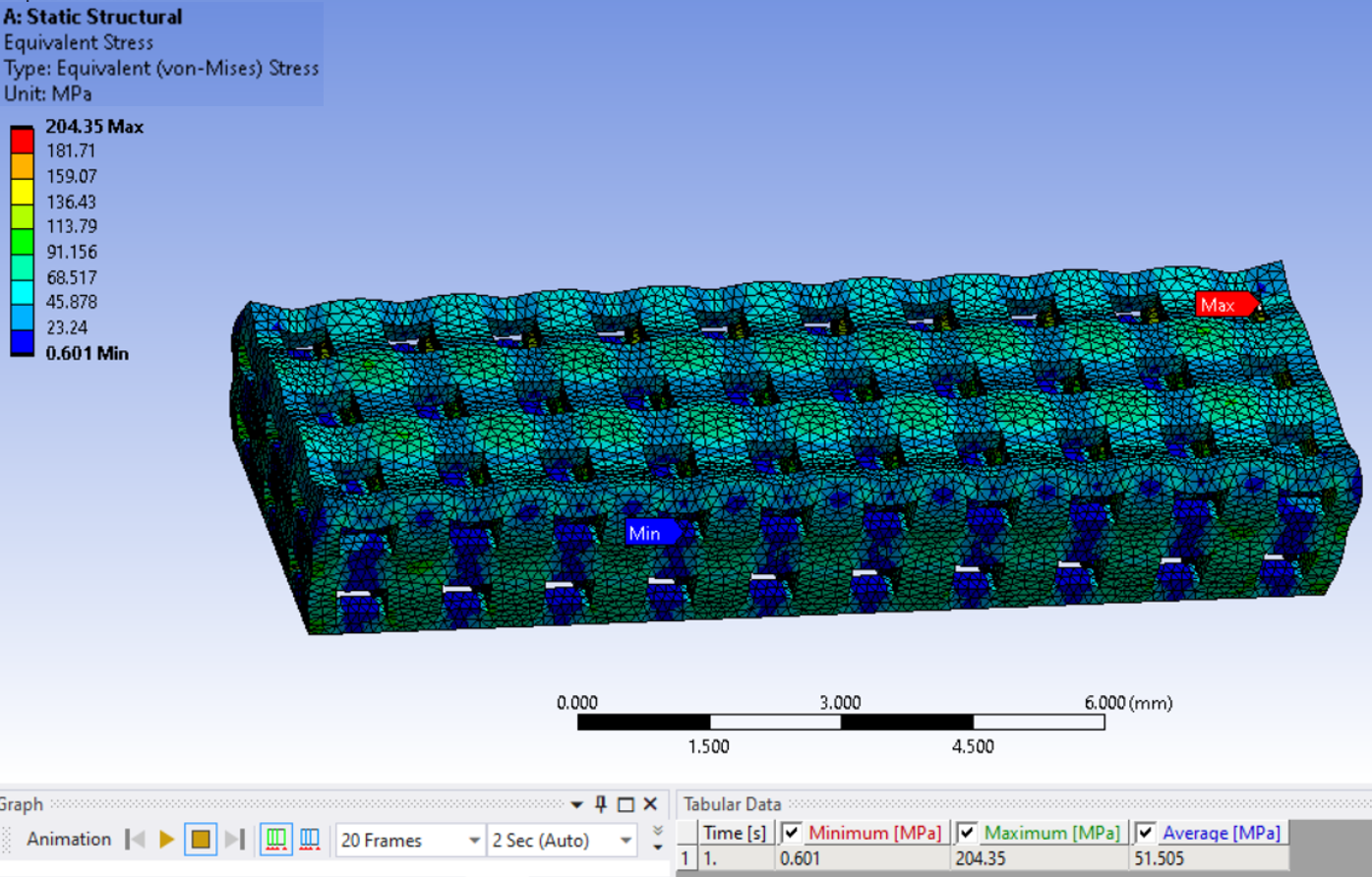 |

References:

1. Zhai Y, Galarraga H, Lados DA: Microstructure, static properties, and fatigue crack growth mechanisms in Ti-6Al-4V fabricated by additive manufacturing: LENS and EBM. *Engineering Failure Analysis* 2016, 69:3-14.
